# Variant surface antigens of malaria parasites: functional and evolutionary insights from comparative gene family classification and analysis

**DOI:** 10.1186/1471-2164-14-427

**Published:** 2013-06-27

**Authors:** Christian Frech, Nansheng Chen

**Affiliations:** 1 Department of Molecular Biology and Biochemistry, Simon Fraser University, 8888 University Drive, Burnaby, B.C V5A 1S6, Canada

**Keywords:** Malaria parasites, Plasmodium, Comparative genomics, Variant surface antigens, Gene family classification, Vaccine target

## Abstract

**Background:**

*Plasmodium* parasites, the causative agents of malaria, express many variant antigens on cell surfaces. Variant surface antigens (VSAs) are typically organized into large subtelomeric gene families that play critical roles in virulence and immune evasion. Many important aspects of VSA function and evolution remain obscure, impeding our understanding of virulence mechanisms and vaccine development. To gain further insights into VSA function and evolution, we comparatively classified and examined known VSA gene families across seven *Plasmodium* species.

**Results:**

We identified a set of ultra-conserved orthologs within the largest *Plasmodium* gene family *pir*, which should be considered as high-priority targets for experimental functional characterization and vaccine development. Furthermore, we predict a lipid-binding domain in erythrocyte surface-expressed PYST-A proteins, suggesting a role of this second largest rodent parasite gene family in host cholesterol salvage. Additionally, it was found that PfMC-2TM proteins carry a novel and putative functional domain named MC-TYR, which is conserved in other *P. falciparum* gene families and rodent parasites. Finally, we present new conclusive evidence that the major *Plasmodium* VSAs PfEMP1, SICAvar, and SURFIN are evolutionarily linked through a modular and structurally conserved intracellular domain.

**Conclusion:**

Our comparative analysis of *Plasmodium* VSA gene families revealed important functional and evolutionary insights, which can now serve as starting points for further experimental studies.

## Background

Malaria is a major health problem in the world. Although global disease incidence is currently on decline, malaria remains responsible for at least 200 million infections and half a million deaths every year, in particular among immune-naïve African children under the age of five [1]. Human malaria is an infectious disease caused by five different species of eukaryotic parasites of the genus *Plasmodium* and is transmitted by Anopheles mosquitoes. *Plasmodium falciparum* is the most prevalent and virulent human malaria parasite accounting for almost all malarial deaths worldwide. *Plasmodium vivax* is the most prevalent malaria parasite outside Africa where despite much lower mortality rates it represents a huge socioeconomic burden in many countries [2]. *Plasmodium ovale* and *Plasmodium malariae* cause a more benign form of human malaria and are responsible for only a small percentage of global infections. *Plasmodium knowlesi*, although traditionally considered a non-human parasite, is responsible for a potentially life-threatening zoonotic form of human malaria acquired from Southeast Asian macaque monkeys [3]. Other *Plasmodium* parasites are important model organisms in malaria research, including *Plasmodium yoelii*, *Plasmodium chabaudi*, and *Plasmodium berghei* (rodent parasites) as well as *Plasmodium gallinaceum* (bird parasite). A tree detailing the phylogenetic relationship of these and other *Plasmodium* species is provided in Additional file 1.

One of the most pressing issues in malaria research is the development of an effective antimalarial vaccine. Unfortunately, despite many decades of research, this goal has yet to be achieved. RTS,S is currently the most promising *P. falciparum* vaccine candidate, but latest results from clinical trials showed that RTS,S provides only modest protection against both clinical and severe malaria in young infants [4,5]. For *P. vivax* the situation looks even grimmer with currently no vaccine candidate in advanced clinical trials [6]. In 2002, publication of the first two *Plasmodium* genomes, *P. falciparum* and *P. yoelii*, promised to revolutionize vaccine development by laying out the complete map of *P. falciparum* genes, including a comprehensive inventory of putative antigens that could serve as vaccine targets [7,8]. This monumental achievement was followed by the publication of the genomes of four additional *Plasmodium* species, including *P. chabaudi* and *P. berghei* in 2005 [9] as well as *P. vivax* and *P. knowlesi* in 2008 [10,11]. The *P. gallinaceum* genome was sequenced to three-fold coverage in 2007 and is currently unpublished. Although the availability of so many *Plasmodium* genome sequences provides now a rich resource for (comparative) genomics studies to learn more about parasite biology and immune evasion strategies [12,13], the promise of an effective antimalarial vaccine has yet to be fulfilled.

One major obstacle in malaria vaccine development is the notorious variability of parasite antigens. These antigens are expressed at the surface of the parasite or of the infected erythrocyte and are typically encoded by large gene families located at subtelomeric regions of chromosomes. We now know that each of the sequenced *Plasmodium* genomes possesses an extensive and often species-specific array of variant surface antigens (VSAs) [14]. The clinically most important and best studied *Plasmodium* VSA gene family is *var*, which encodes for about 60 erythrocyte surface-expressed proteins known as *P. falciparum* erythrocyte membrane protein 1 (PfEMP1) [8]. In a process called antigenic variation, *var* gene expression switches from one family member to another over the course of an infection, allowing the parasite to evade the host immune system and to establish a chronic infection [15]. In addition, PfEMP1 mediates adherence of infected erythrocytes to both uninfected erythrocytes and endothelial cells, which is responsible for the most severe clinical complications of *P. falciparum* malaria and makes PfEMP1 the prime virulence factor in this species.

Besides PfEMP1, *Plasmodium* parasites express many additional VSAs throughout their complex life cycle. In *P. falciparum*, this includes the largest *P. falciparum* gene family *rif/stevor* (~190 genes) [8,16], the *surfin* gene family (10 genes) [17], and *Pfmc-2TM* (12 genes) [18]. In *P. vivax*, the largest gene family by far is *vir* (~300 genes) [10,19], which is related to homologous VSA gene families named *kir* (65 genes) in *P. knowlesi*[11,20] and *yir* (~800 genes), *bir* (~100 genes), and *cir* (~200 genes) in *P. yoelii*, *P. berghei* and *P. chabaudi*, respectively [7,9,21]. Together, these five gene families form the large *pir* superfamily, the largest known gene family in *Plasmodium* parasites [20]. To date no *pir* genes have been identified in *P. falciparum*. *P. knowlesi* possesses an additional large VSA gene family named *SICAvar* (28 genes), the first *Plasmodium* gene family demonstrated to undergo antigenic variation [11,22]. In rodent malaria parasites the second largest VSA gene family after *pir* is *pyst-a*, which in primate parasites consists of only a single member suggesting extensive expansion of this family in the rodent malaria species [7]. Besides VSAs, *Plasmodium* genomes encode another large repertoire of proteins termed the ‘exportome’ that is of potential interest for vaccine development [23]. Proteins in this set carry an N-terminal sequence motif termed *Plasmodium* export element (PEXEL) or vacuolar transport signal (VTS) that targets these proteins beyond the parasitophorous vacuole to the cytosol of the infected erythrocyte [24,25]. Exported proteins are then trafficked further to the erythrocyte surface or remain in the cytosol to help remodeling the infected host cell. Like VSAs, exported proteins are typically organized into subtelomeric variant gene families, most of which are species subset-specific [23]. Probably the most prominent exported gene family is the large and highly divergent gene family *phist*, which has ~40-100 known members in each of the three primate parasites but only a single known member in rodent parasites [23].

Although their large numbers suggest that VSAs and exported proteins are of major importance for the parasite, we currently know surprisingly little about their biological functions. For example, proposed roles for PIR proteins include antigenic variation, immune evasion, signaling, trafficking, protein folding, and adhesion, but direct evidence for any PIR function is still lacking [14]. Similarly, apart from expression and localization information, the exact functions of PfMC-2TM, SURFIN, PYST-A, and PHIST proteins are currently unknown. Furthermore, the *P. falciparum* genome contains over a dozen exported gene families named *hyp1* to *hyp17* whose functions have yet to be determined. The difficulty in elucidating the function of these gene families is due in part to the presence of many functionally redundant paralogs, which makes gene knockout studies challenging. In such cases it would help if one can identify and work with low copy number orthologous gene families in more accessible model parasites. Besides unknown functions, the evolutionary history of many *Plasmodium* variant gene families is also poorly understood. For example, standard sequence similarity searches reveal no obvious homologs for the major surface antigens of *P. falciparum* (PfEMP1) and *P. knowlesi* (SICAvar) outside their respective species, raising the question about their evolutionary origin. Similarly, there are currently no known *pir* homologs in *P. falciparum*, although *rif/stevor* has been suggested as related gene family based on shared sequence motifs and secondary structural features [20]. The identification of functional homologs of VSAs across *Plasmodium* species is important because it aids comparative immunological studies, gives new insights into the evolutionary adaptation of malaria parasites to their respective hosts, and provides a means to transfer functional annotations from model to human parasites and *vice versa*.

In this study, we use a recently developed comparative gene family classification strategy [26] to classify VSAs and exported proteins across seven *Plasmodium* genomes, including *P. falciparum*, *P. vivax*, *P. knowlesi*, *P. yoelii*, *P. chabaudi*, *P. berghei*, and *P. gallinaceum*. We hypothesized that the sensitive sequence-based clustering of the entire body of currently available *Plasmodium* proteins will yield new insights into VSA function and evolution, which in turn could open up new avenues for vaccine development. In this strategy, protein sequences from *Plasmodium* genomes are first clustered into a hierarchical tree using average-linkage clustering and the resulting tree is then searched for clusters corresponding to known VSA gene families. Finally, identified clusters are closely analyzed for gene content and inter-cluster relationships. This analysis resulted in several noteworthy findings, including the identification of unusually well conserved PIR orthologs that are of potential interest for vaccine development, prediction of the likely function of PYST-A proteins, discovery of a novel and putatively functional PfMC-2TM domain named MC-TYR, new conclusive evidence supporting the common evolutionary origin of PfEMP1, SICAvar, and SURFIN proteins, and the identification of many new VSA gene family members, including new *phist* gene family members in rodent parasites. Collectively, these findings provide vital starting points for future experimental studies.

## Results

### Curation and comparative classification of variant gene families in *Plasmodium* genomes

We curated VSA and exported gene families by reviewing the literature as well as gene annotations in PlasmoDB 7.0 [27]. We were interested in all *Plasmodium* gene families that met one of the following criteria: (a) expressed at the parasite surface or the surface of infected erythrocytes; (b) predicted host cell localization by virtue of the presence of a PEXEL/VTS export motif; any other gene family that is (c) species (subset)-specific or (d) located at subtelomeric regions of chromosomes. Using these criteria, we compiled a list of 59 gene families (Table 1 and Additional file 2), which from now on we refer to as *Plasmodium variant gene families*.

**Table 1:** ***Plasmodium *** **variant gene families and classification performance**

**Gene family**	**Sn**	**Sp**	**Jacc**	**pfal**	**pviv**	**pkno**	**pyoe**	**pber**	**pcha**	**pgal**
**VSA**										
*var*	1.00	0.99	0.99	67 (66)	0	0	0	0	0	0
*vir-kir*	0.94	0.82	0.78	1	310 (262)	66 (65)	3	2	2	1
*yir-bir-cir*	0.99	0.98	0.97	0	0	0	897	100 (99)	195 (191)	0
*rif/stevor*	0.99	0.98	0.98	192 (190)	0	0	0	0	0	0
*SICAvar*	1.00	0.90	0.90	0	0	31 (28)	0	0	0	0
*Pfmc-2TM*	1.00	1.00	1.00	12 (12)	0	0	0	0	0	0
*TryThrA-PvTRAG*	1.00	1.00	1.00	4 (4)	36 (36)	26	5	7	6	1
*surfin/Pvstp1*	1.00	1.00	1.00	9 (9)	2 (2)	0	0	0	0	2
*cys6*	1.00	1.00	1.00	10 (10)	11	12	14	10	10	6
*P25_28*	1.00	1.00	1.00	2 (2)	2 (2)	2 (2)	2 (2)	2 (2)	2 (2)	1
*PcEMA1*	0.71	0.92	0.67	0	0	0	2	1	13 (17)	0
*pyst-a*	1.00	0.90	0.90	1	1	1	169 (138)	23 (22)	132 (131)	1
**VSA (invasion-linked)**										
*dbl*	1.00	1.00	1.00	5 (5)	2 (2)	4	2	2	2	1
*rbp/235 kDa*	1.00	1.00	1.00	5 (5)	8 (8)	1	19	6	8	0
*msp-3*	0.89	0.89	0.80	4 (6)	14 (12)	4	2	2	2	0
*msp-7*	1.00	1.00	1.00	6 (6)	11 (11)	4	3	3	3	1
*rhoph1/clag*	1.00	1.00	1.00	5 (5)	3 (3)	1 (1)	2	2	3	1
*TRAP*	0.60	0.75	0.50	4 (5)	4	3	4	4	4	3
*TSP_1*	1.00	0.73	0.73	8 (6)	8 (7)	7 (5)	9 (5)	8 (5)	8 (7)	5
*PPLP*	1.00	1.00	1.00	5 (5)	5	5	5	5	5	3
**Exported**										
*phist/rad*	0.97	0.89	0.86	72 (66)	74	43	2	2	2	4
*gbp130*	1.00	1.00	1.00	3 (3)	0	0	0	0	0	0
*pst-a*	0.97	1.00	0.97	10 (10)	9 (10)	6	12 (12)	4	28	1
*emp3*	0.50	1.00	0.50	1 (2)	1	1	0	0	0	0
*ab_hyda*	1.00	1.00	1.00	4 (4)	2	2	2	2	2	1
*ab_hydb*	1.00	1.00	1.00	4 (4)	1	1	0	0	0	1
*HRP*	1.00	1.00	1.00	2 (2)	0	0	0	0	0	0
*hyp1*	0.50	1.00	0.50	1 (2)	0	0	0	0	0	0
*hyp2*	0.50	0.50	0.33	2 (2)	0	0	0	0	0	0
*hyp4*	1.00	1.00	1.00	9 (9)	0	0	0	0	0	0
*hyp5*	1.00	0.89	0.89	9 (8)	0	0	0	0	0	0
*hyp6*	1.00	0.40	0.40	5 (2)	0	0	0	0	0	0
*hyp7*	1.00	1.00	1.00	3 (3)	0	0	0	0	0	0
*hyp8*	1.00	1.00	1.00	2 (2)	0	0	0	0	0	0
*hyp9*	1.00	1.00	1.00	5 (5)	0	0	0	0	0	0
*hyp10*	1.00	1.00	1.00	2 (2)	0	0	0	0	0	0
*hyp11*	1.00	1.00	1.00	5 (5)	6	5	1	1	1	0
*hyp12*	1.00	0.75	0.75	4 (3)	0	0	0	0	0	0
*hyp13*	1.00	1.00	1.00	2 (2)	0	0	0	0	0	0
*hyp15*	0.75	0.75	0.60	4 (4)	0	0	0	0	0	0
*hyp16*	1.00	1.00	1.00	2 (2)	0	0	0	0	0	0
*hyp17*	1.00	0.67	0.67	3 (2)	0	0	0	0	0	0
*pk-fam-b*	1.00	0.83	0.83	0	1	12 (10)	0	0	0	0
*pk-fam-c*	1.00	1.00	1.00	0	0	5 (5)	0	0	0	0
*pk-fam-e*	1.00	1.00	1.00	0	0	3 (3)	0	0	0	0
**Other (species-specific or sub-telomeric)**										
*etramp*	0.97	0.94	0.91	15 (14)	10 (9)	9	15	6 (7)	12	4
*acs*	1.00	0.93	0.93	14 (13)	5	5	5	5	7	5
*ACBP*	1.00	1.00	1.00	4 (4)	0	0	0	0	0	0
*pv-fam-g*	1.00	1.00	1.00	3	3 (3)	3	3	3	3	3
*pk-fam-a*	1.00	0.89	0.89	0	0	9 (8)	0	0	0	0
*pc-fam*	0.85	1.00	0.85	0	0	0	5	1	17 (20)	0
*pk-fam-d*	0.50	1.00	0.50	0	0	1 (2)	0	0	0	0
*pv-fam-d*	1.00	0.57	0.57	1	28 (16)	9	0	0	0	0
*pyst-c*	1.00	0.82	0.82	0	0	0	22 (18)	3	11	0
*pv-fam-b*	1.00	1.00	1.00	0	6 (6)	1	0	0	0	0
*pv-fam-c*	1.00	1.00	1.00	0	7 (7)	0	0	0	0	0
*pyst-d*	0.77	1.00	0.77	0	0	0	10 (13)	0	0	0
*pyst-b*	1.00	0.96	0.96	0	0	0	56 (54)	28	21	0
*pv-fam-h*	1.00	0.80	0.80	4	5 (4)	3	0	0	0	0
**Average**	**0.94**	**0.92**	**0.87**							

Fifty-nine *Plasmodium* variant gene families were curated from the literature and public databases and categorized as follows: (a) VSAs (11 families); (b) VSAs linked to erythrocyte invasion (8 families); (c) predicted to be exported to the host erythrocyte (25 families); (d) other subtelomeric or species-specific gene families (15 families). Better studied gene families are shown at the top of each category. Classification performance of each gene family is measured in terms of sensitivity (Sn), specificity (Sp), and Jaccard index (Jacc). The remaining columns show how many genes of each species have been classified to belong to this gene family (excluding annotated pseudogenes and gene fragments). Numbers in parentheses indicate how many genes of each species served as reference gene family members. A more comprehensive version of this table including numbers of identified genes in non-*Plasmodium* species is available in Additional file 2. Abbreviations: VSA… variant surface antigen; pfal… *P. falciparum*; pviv… *P. vivax*; pkno… *P. knowlesi*; pyoe… *P. yoelii*; pber… *P. berghei*; pcha… *P. chabaudi*; pgal… *P. gallinaceum.*

After curation, we classified *Plasmodium* variant gene families using a comparative gene family classification strategy described previously [26]. Briefly, we hierarchically clustered the combined set of all protein sequences with MC-UPGMA [28] and extracted those sub-trees that maximally overlapped with the curated gene families. Most of the 59 curated gene families were resolved with both high sensitivity (Sn) and specificity (Sp) (Table 1). Average and median Jaccard indices (J) of extracted clusters were 0.87 and 0.98, respectively. Almost half of gene families (28 gene families, 47%) were clustered perfectly with a Jaccard index of 1.0. An additional 20 gene families (34%) clustered with a good Jaccard index ≥ 0.75. Only 11 gene families clustered with lower quality (J < 0.75) due to low sensitivity (*PcEMA1*, *emp3*, *hyp1*, *pk-fam-d*), low specificity (*TSP_1*, *hyp17*, *pv-fam-d*, *hyp6*) or both (*hyp15*, *TRAP*, *hyp2*). Overall, we conclude that our clustering strategy works well on *Plasmodium* variant gene families and produces high-quality protein sequence clusters.

### PIR contains single ultra-conserved ortholog in each *Plasmodium* species

The large *pir* gene family separates nicely into two large non-overlapping clusters of high sensitivity and specificity (Table 1). One cluster represents the *vir* and *kir* subfamilies of *P. vivax* and *P. knowlesi*, respectively (384 genes; Sn = 94%; Sp = 82%; J = 78%), and the other cluster represents the *yir*, *bir*, and *cir* subfamilies from *P. yoelii*, *P. berghei*, and *P. chabaudi*, respectively (1,192 genes; Sn = 99%; Sp = 98%; J = 97%). The somewhat reduced specificity of the *vir/kir* gene cluster is due to inclusion of the *pv-fam-c* gene family (7 genes) that likely represents a *bona fide vir* subfamily (see below). In addition, the *vir/kir* cluster contains 59 *P. vivax* hypothetical proteins, many of which carry a predicted *vir* domain and are thus likely true but currently unannotated members of the *vir* gene family (see annotated gene models of the complete *vir* gene cluster at http://genome.sfu.ca/projects/gfc-plasmodium/).

**Figure 1:**
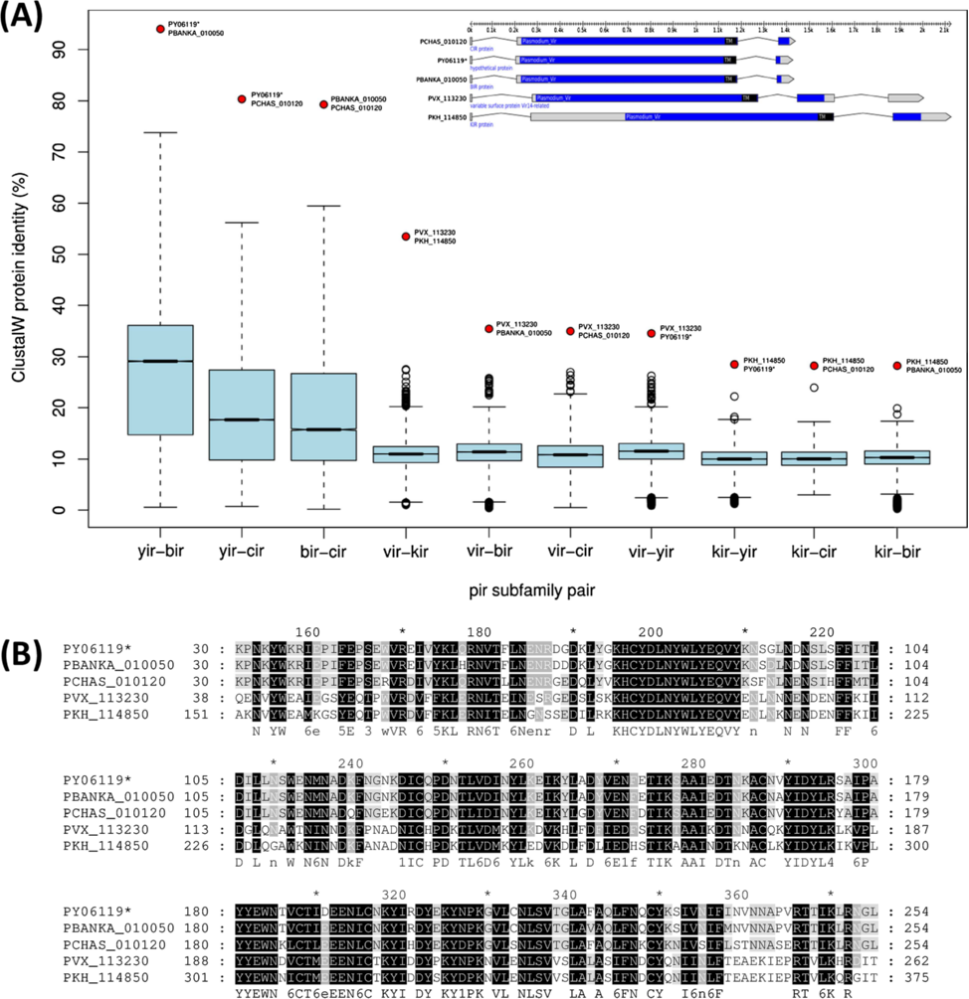
**Identification of ultra-conserved PIR orthologs. (A)** PIR protein sequences from five *Plasmodium* species, including *P. yoelii* (826 *yir* genes), *P. berghei* (135 *bir* genes), *P. chabaudi* (196 *cir* genes), *P. vivax* (345 *vir* genes), and *P. knowlesi* (68 *kir* genes), were globally aligned in an all-*vs.*-all pairwise manner using ClustalW. Box plots show the resulting distribution of global percent identity values for each pair of species. For each species pair, one outlier (red circles) representing a pair of proteins with increased cross-species conservation levels is identified, including PY06119 (*P. yoelii*), PBANKA_010050 (*P. berghei*), PCHAS_010120 (*P. chabaudi*), PVX_113230 (*P. vivax*), and PKH_114850 (*P. knowlesi*). The inset shows the gene models of these five proteins with the predicted *Plasmodium_vir* domain (PF05795) indicated in blue and predicted transmembrane domains (TM) indicated in black. The second exon of PY06119 was found to be truncated and an improved version of this gene model (PY06119*) was therefore used in the analysis. **(B)** Multiple sequence alignment of the five conserved PIR orthologs. Note the exceptional high degree of sequence conservation (indicated by black and gray columns), suggesting an important molecular function for this particular PIR protein in each *Plasmodium* species.

To our surprise we found three rodent PIR proteins nested deep within the *vir/kir* cluster instead of clustering with their respective paralogs (Additional file 3). This unexpected result suggested that some of the otherwise highly divergent PIR proteins are well conserved across species. Subsequent comprehensive analysis of PIR sequence conservation revealed that each of the five *Plasmodium* species contains a single and likely orthologous PIR protein that is clearly better conserved between species than any other member of the PIR family (Figure 1A). The five genes are: *P. vivax* Vir14-related protein PVX_113230; *P. knowlesi* KIR protein PKH_114850; *P. yoelii* hypothetical protein PY06119; *P. berghei* BIR protein PBANKA_010050; and *P. chabaudi* CIR protein PCHAS_010120. Orthology of these five PIR proteins is supported by OrthoMCL DB (version 5.0, orthologous group OG5_173782) [29] and by the fact that gene synteny is conserved among the five species (PlasmoDB 9.3). Multiple sequence alignment of the five conserved PIR orthologs reveals a 224 aa long, gapless block recognized as the *Plasmodium_Vir* domain (PF05795). The block spans 56% of the average sequence length and has 107 columns (48%) perfectly conserved (Figure 1B). This exceptional high degree of sequence conservation within an otherwise highly divergent gene family suggests that these genes are direct descendants of the founder member of the large *pir* gene family and that they serve an ancestral and probably special function in malaria parasites.

Identification of the likely prototypical members of the large *pir* gene family allowed us to re-examine whether there exists conserved PIR homologs outside *P. vivax/P. knowlesi* and rodent malaria parasites, in particular within the Laverania clade including *P. falciparum*. NCBI PSI-BLAST search (http://blast.ncbi.nlm.nih.gov) seeded with the five conserved PIR orthologs did not reveal significant sequence similarity within *P. falciparum* or any other species not currently known to contain PIR proteins. Similarly, multiple rounds of Jackhmmer searches [30] seeded with the alignment from Figure 1 did not identify convincing homologs. Thus, despite extensive sequence conservation between the primate and rodent parasites, PIR is apparently not conserved in other *Plasmodium* or Apicomplexan species, suggesting that PIR surface antigens perform an important function unique to the *P. vivax/P. knowlesi* and rodent clade of malaria parasites.

### PYST-A proteins predicted to be involved in lipid binding and transfer

Gene family expansion is an important genomic process by which parasites adapt to different lifestyles and host environments [31,32]. Our classification strategy readily identifies differentially expanded gene families in several *Plasmodium* species (Table 1), including *rhoph1/clag*, *surfin* and *acs* (expanded in *P. falciparum*), *msp-3*, *msp-7*, and *pv-fam-d* (expanded in *P. vivax*), *TryThrA/PvTRAG* (expanded in *P. vivax* and *P. knowlesi*), *phist* and *hyp11* (expanded in the three primate parasites), *rbp/235 kDa* and *pyst-c* (expanded in *P. yoelii*), and *PcEMA1*, *pc-fam*, and *pst-a* (expanded in *P. chabaudi*).

One of the most striking examples of a differentially expanded gene family in *Plasmodium* parasites is the *P. yoelii* gene family *pyst-a* (Table 1). *Pyst-a* has been shown to contain only a single member in primate malaria parasites but over one hundred members in *P. yoelii* and *P. chabaudi*[7,9]. In our analysis, PYST-A proteins cluster almost perfectly (Sn = 100%; Sp = 90%; J = 90%) within a larger cluster containing also PC-FAM-1 and PB-FAM-1 proteins, clearly showing that these three gene families are equivalent (orthologous) in the three rodent parasite species. Total gene numbers in the combined *pyst-a/pc-fam-1/pb-fam-1* sequence cluster are 168, 132, 23 genes in *P. yoelii*, *P. chabaudi*, and *P. berghei*, respectively. As expected, we found only a single *pyst-a* gene family member in primate malaria parasites (PF14_0604 in *P. falciparum*, PVX_117290 in *P. vivax*, and PKH_124210 in *P. knowlesi*). A single copy of *pyst-a* was also detected in the bird parasite *P. gallinaceum*, supporting the idea of extensive proliferation of the *pyst-a* gene family in the rodent malaria parasite lineage [7].

**Figure 2:**
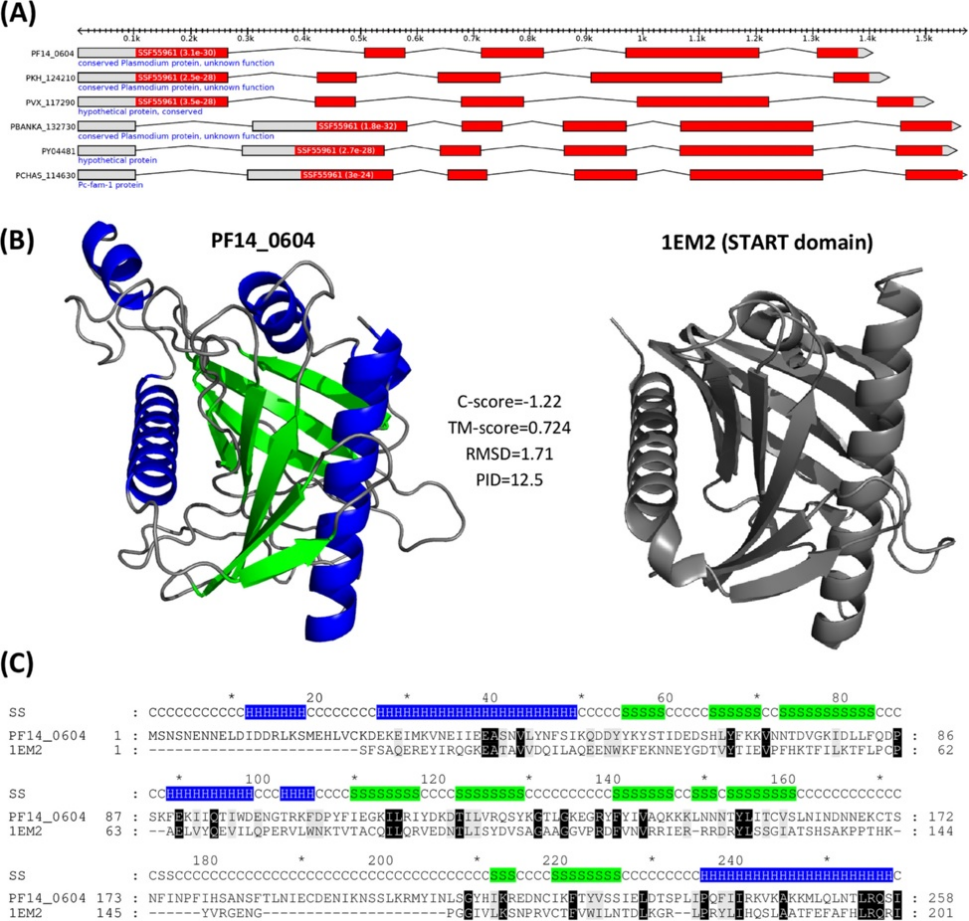
**PYST-A proteins predicted to be involved in lipid binding and transfer. (A)** Predicted *Bet v1-like superfamily* domain (SSF55961, in red) in selected *pyst-a* gene family members from six *Plasmodium* species. Hmmer3 E-values shown in parentheses. **(B)** I-TASSER predicted homology model (C-score = −1.22) of the single *P. falciparum pyst-a* gene family member PF14_0604 (left) next to crystal structure of steroidogenic acute regulatory-related lipid transfer (START) domain-containing human protein MLN64 (PDB entry 1EM2; right). Predicted alpha helices and beta strands of PF14_0604 highlighted in blue and green, respectively. Note the overall high similarity between the two structures (TM-score = 0.724; RMSD = 1.71) **(C)** Protein sequence alignment obtained by threading PF14_0604 onto 1EM2 using MUSTER. Percent sequence identity (PID) of structurally aligned residues is 12.5% over 78% of PF14_0604 sequence length. Secondary structure (SS) elements colored according to structural model in B. Identical and similar amino acids colored in black and gray, respectively. Sequence and structural similarity suggest a potential role of the *pyst-a* gene family in lipid binding and transfer.

Interestingly, while examining annotated *pyst-a* gene models (Figure 2A), we noticed that most *pyst-a* gene family members carry a predicted *Bet v1-like superfamily* domain [33] (http://supfam.org/SUPERFAMILY/cgi-bin/scop.cgi?ipid=55961; HMMER3 E-value ≤ 1e-10), suggesting a role of PYST-A proteins in lipid transport. In search for further bioinformatics support for this possibility, we used I-TASSER to predict the tertiary and secondary structure of *P. falciparum* gene family member PF14_0604 (Figure 2B and Figure 2C). The predicted protein structure is very similar to several human proteins carrying the *steroidogenic acute regulatory-related lipid transfer* (START) domain, including PDB protein 1EM2 (C-score = −1.22; TM-score = 0.724; RMSD = 1.71). Taken together, our bioinformatics analysis suggests that the second largest rodent malaria parasite gene family *pyst-a* is involved in lipid binding and transport, probably playing a role in host cholesterol salvage (see Discussion).

### PfMC-2TM proteins are conserved in rodent parasites and related to HYP8 and HYP2 through the novel MC-TYR domain

Hierarchical protein sequence clustering is a powerful approach to elucidate distant evolutionary relationships between gene families [34]. Table 2 summarizes known and novel evolutionary links between *Plasmodium* variant gene families as suggested by hierarchical clustering. Novel links of note include links between *Pfmc-2TM* and *hyp8* (BLAST match pair percentage (MP) = 100%; average E-value of BLAST match pairs (E) = 1.6), *Pfmc-2TM/hyp8* and *hyp2* (MP = 76.7%; E = 5.6), *hyp4* and *hyp6* (MP = 80%, E = 1.9), *hyp5* and *hyp15* (MP = 100%, E = 0.082), *hyp16*, *pv-fam-h*, and *pk-fam-c* (MP = 30.6%, E = 0.72), as well as *pk-fam-a*, *pk-fam-b* and *pv-fam-d* (MP = 11.6%, E = 0.048). Thus, several *Plasmodium* variant gene families currently assumed to be species-specific have in fact putative members in other *Plasmodium* species, opening up new avenues for elucidating gene function. Of note, we found that *pv-fam-c* is entirely nested within the larger *vir/kir* cluster (MP = 2.9%, E = 1.3) and thus likely represents a novel *vir* subfamily that we named *vir36*. *pyst-d* (InterPro IPR006492), a family of ~15 short (median length 60 aa) *P. yoelii* proteins, was found to be completely nested within the large *pir* gene family. Inspection of *pyst-d* and neighboring gene models revealed that this gene family is likely spurious and consists entirely of misannotated exons belonging to adjacent *yir* gene models. Other novel links in Table 2 are of lower connectivity and should be regarded as tentative.

**Table 2:** **Established and novel evolutionary links between *Plasmodium *** **variant gene families predicted by hierarchical clustering**

**Cluster A**	**Size A**	**Cluster B**	**Size B**	**MP#**	**MP%**	**Avg. Evalue**	**Avg. PID(Stddev)**
**Established links**							
*rif*	161	*stevor*	31	281,244	74.8%	0.016	26.4(3.1)
*vir/kir*	385	*yir/bir/cir*	1,192	2,087	0.4%	9.7	28.2(5.2)
*surfin*	15	*Pvstp1*	2	90	93.8%	3.9e-15	38.3(6.6)
*var*	67	*dbl*	18	707	42.8%	1.7e-10	25.0(2.9)
*phist-c*	80	*rad*	58	718	15.5%	0.38	23.9(3.7)
*phist-c/rad*	138	*phist-b*	21	241	8.3%	2.4	25.2(4.2)
*phist-c/rad/phist-b*	168	*phist-a*	29	273	4.0%	1.6	26.4(5.3)
*ab_hydb*	10	*pst-a*	77	787	99.6%	1.7e-11	33.1(6.2)
*ab_hydb/pst-a*	87	*ab_hyda*	17	231	10.4%	3.8	28.4(5.5)
*surfin/Pvstp1*	17	*SICAvar*	31	2,003	35.7%	0.013	37.8(8.4)
*surfin/Pvstp1/SICAvar*	51	*var*	67	435	1.8%	1.9	37.9(6.8)
**Novel links**							
*surfin/Pvstp1/SICAvar/var*	149	*pir*	1,814	772	0.1%	14	30.5(6.5)
*Pfmc-2TM*	12	*hyp8*	2	24	100.0%	1.6	27.9(2.4)
*Pfmc-2TM/hyp8*	14	*hyp2*	2	23	76.7%	5.6	32.6(2.8)
*hyp4*	9	*hyp6*	5	40	80.0%	1.9	26.7(1.5)
*hyp15*	4	*hyp5*	9	44	100.0%	0.082	31.0(5.5)
*pk-fam-a*	9	*pk-fam-b*	13	38	16.2%	2.3e-06	55.7(18.4)
*pk-fam-a/pk-fam-b*	22	*pv-fam-d*	38	137	11.6%	0.048	30.6(7.9)
*pyst-a/pc-fam-1/pb-fam-1*	328	*PcEMA1*	16	90	1.5%	7.7e-32	50.6(8.9)
pv-fam-h*	12	*hyp16*	2	16	80.0%	7.8e-12	33.7(6.8)
*hyp16/pv-fam-h*	12	*pk-fam-c*	5	22	30.6%	0.72	38.1(5.5)
*TSP_1*	53	*P25/28*	13	29	1.1%	18	32.0(11.2)
*hyp11*	19	*rbp/235 kDa*	47	13	1.1%	31	30.4(6.0)
phist-c/rad/phist-b*	168	*pk-fam-e*	3	30	4.5%	1.9e-06	67.5(14.3)
vir/kir*	385	*pv-fam-c*	7	94	2.9%	1.3	27.0(5.2)
pir*	1,814	*pyst-d*	10	179	0.9%	0.11	64.2(14.6)

The hierarchical tree was searched for neighboring and parental clusters where both clusters (denoted A and B) map to variant gene families as identified in Table 1. Such cluster pairs are shown if (a) their BLAST match pair percentage (*MP%*) is ≥ 1% or (b) they share at least 100 BLAST match pairs (*MP#*). *Avg. E-value* and *Avg. PID* denote the average E-value and percent identity of all BLAST match pairs between the two clusters. *Size A* and *Size B* indicate numbers of genes in respective clusters, excluding annotated pseudogenes and gene fragments. An asterisk next to cluster A indicates that it is a parental cluster of cluster B, *i.e.* cluster B is fully contained in cluster A. Our search predicts both previously established (top) and novel evolutionary links (bottom) between *Plasmodium* variant gene families.

**Figure 3:**
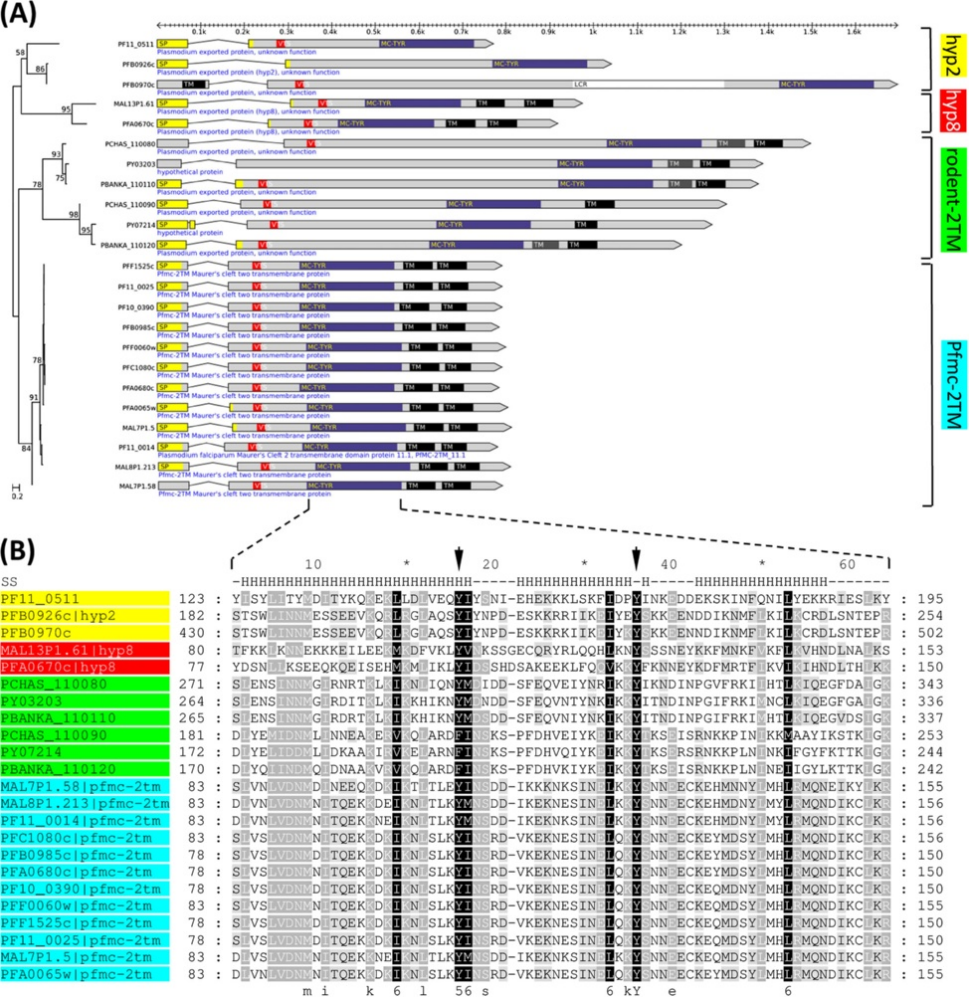
**PfMC-2TM is conserved in rodent malaria parasites and related to *P. falciparum *HYP2 and HYP8 proteins through the novel MC-TYR domain. (A)** Members of the *P. falciparum* gene families *Pfmc-2TM*, *hyp8* and *hyp2* as well as six rodent parasite genes share a conserved ~70 aa domain named MC-TYR (dark blue) located between the PEXEL/VTS motif (shown in red) and the two C-terminal transmembrane domains (TM, shown in black). TM domains in dark gray are predicted by Phobius but not TMHMM. SignalP predicted signal peptides (SP) shown in yellow. The phylogenetic tree to the left is a bootstrap consensus tree based on the MC-TYR alignment shown in panel **B**. Numbers represent bootstrap values (values below 60 not shown). **(B)** T-coffee multiple sequence alignment of MC-TYR. The name stands for *Maurer’s clefts tyrosine* domain due to two almost invariably conserved tyrosine residues (Y, indicated by arrows), which are of potential functional importance. Top row: ‘H’ denotes residues within predicted alpha helices, thus MC-TYR has a predicted three alpha-helical secondary structure (SS).

*Pfmc-2TM* is an actively studied *P. falciparum* VSA gene family [18,35,36], which motivated us to investigate its putative link with the two *P. falciparum* hypothetical gene families *hyp8* and *hyp2* in detail. The 12 annotated PfMC-2TM proteins cluster first with two *P. falciparum* gametocyte-exported proteins of the *hyp8* gene family (MAL13P1.61/GEXP07 and PFA0670c/GEXP10) and then with a cluster of three *P. falciparum* exported proteins of unknown function, one of which is a member of the *hyp2* gene family (PFB0926c). The second annotated *hyp2* gene family member (PF10_0024) is not found in this cluster and instead groups with *hyp16* and *pv-fam-h* proteins, supporting the previously expressed idea that *hyp2* does not constitute a real gene family [23]. The combined *Pfmc-2TM*/*hyp8*/*hyp2* cluster contains a total of 23 proteins and is shown in Figure 3A. Notably, this cluster also includes six rodent parasite genes of unknown function that share the typical two-exon gene structure with *Pfmc-2TM* genes, including a signal peptide on the short first exon and a conserved PEXEL-like motif (RxLxQ) on the 5′ end of the larger second exon. Most importantly, Phobius [37] predicts two adjacent C-terminal transmembrane (TM) domains for four of the six rodent parasite proteins (posterior probability > 0.2) as well as traces of a second TM domain in the remaining two (posterior probability < 0.2). No TM domains are present in the three *P. falciparum* proteins clustering with (and including) HYP2 protein PFB0926c.

Further inspection of the *Pfmc-2TM*/*hyp8*/*hyp2* gene cluster using multiple protein sequence alignments identified a hitherto unknown ~70 aa long conserved domain located between the PEXEL motif and the two TM domains (Figure 3B). The domain has a predicted three-alpha helical structure and contains two almost invariant tyrosine residues (Y) of potential functional importance. Only in three proteins, one of the two tyrosine residues is conservatively replaced with phenylalanine (F). Based on this observation, we name this novel domain the *Maurer’s clefts tyrosine* (MC-TYR) domain.

To explore the possibility that MC-TYR is conserved beyond the seed cluster shown in Figure 3, we carried out sensitive profile-based searches using PSI-BLAST. Five rounds of NCBI PSI-BLAST initiated with any one of the 12 *Pfmc-2TM* MC-TYR protein sequences consistently identified three additional *P. falciparum* hits, albeit above PSI-BLAST’s inclusion threshold (E > 0.005). These three hits include one of three annotated HYP12 proteins (MAL7P1.6; E = 0.2) and two other exported proteins of unknown function (PF11_0511 and MAL8P1.206; E ≤ 2.7). Like the three proteins of the *hyp2* cluster, these three genes share the typical two-exon gene structure with *Pfmc-2TM* genes except for the TM domains. This data suggests that MC-TYR is likely conserved in additional exported *P. falciparum* proteins and probably includes the *P. falciparum hyp12* gene family. We should mention that neither protein sequence clustering nor PSI-BLAST searches identified a relationship of PfMC-2TM with HYP7, PYST-B or virD proteins, which has been proposed previously based on shared gene architectural features [18,38].

Taken together, we provide first evidence that PfMC-2TM proteins carry a novel and putative functional domain named MC-TYR. MC-TYR is conserved in other *P. falciparum* exported proteins and additional *Plasmodium* species, opening up new opportunities to experimentally characterize the function of this important VSA gene family.

### PfEMP1, SICAvar, and SURFIN are interrelated through a modular and structurally conserved intracellular tryptophan-rich domain

**Figure 4:**
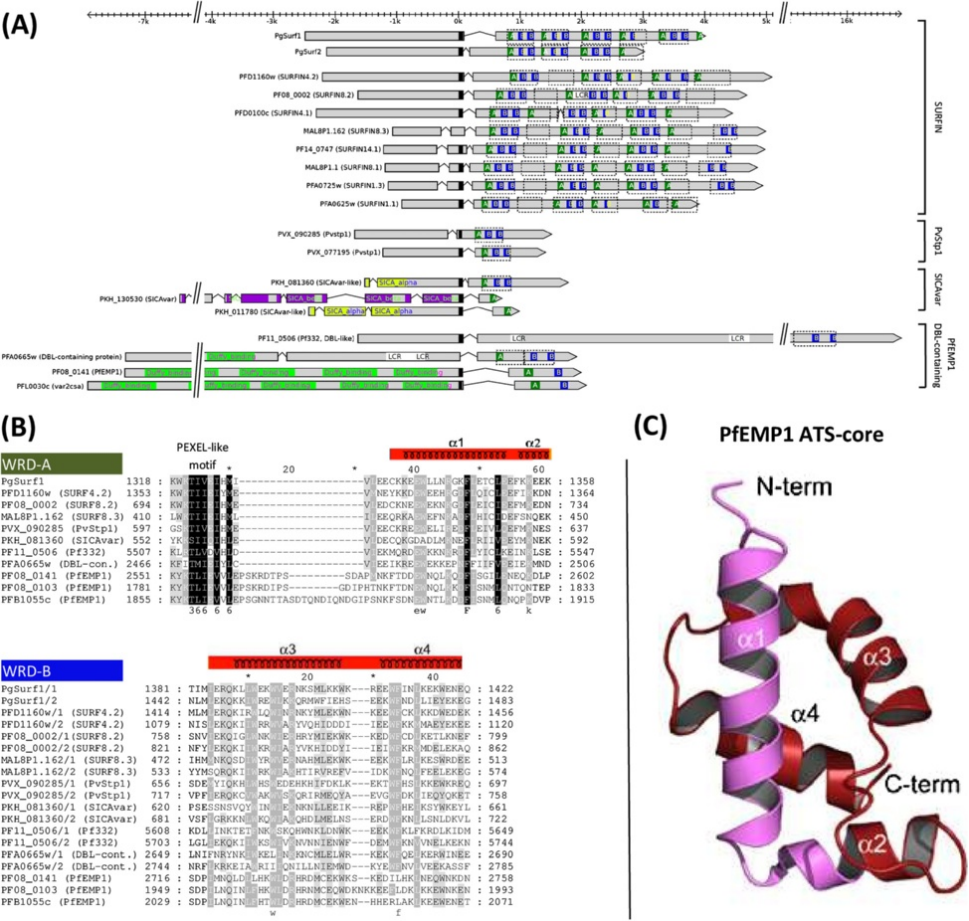
**PfEMP1, SURFIN/PvSTP1, SICAvar and two *P. falciparum *DBL-containing proteins are interrelated through the modular and structurally conserved intracellular tryptophan-rich domain (WRD). (A)** Intracellular regions (right of aligned black transmembrane domains) of SURFIN, PvSTP1, SICAvar, PfEMP1, and two DBL-containing proteins carry 1–7 copies of WRDs (dashed rectangles), which themselves are composed of a variable number of conserved WRD-A (green) and WRD-B (blue) subdomains. Note conservation of complete WRDs (consisting of one **A** and two **B** subdomains) in *P. knowlesi* SICAvar-like gene PKH_081360 and in the bird parasite *P. gallinaceum* (*PgSurf1* and *PgSurf2*). A dashed rectangle extending beyond subdomains indicates that the WRD but not all its subdomains reached statistical significance (E ≤ 0.01) in the Hmmer search. **(B)** Multiple sequence alignment of selected sequences representing the WRD-A (top) and WRD-B (bottom) subdomains. “/1” and “/2” after protein IDs denote first and second occurrences of a subdomain within a (not necessarily the same) WRD, respectively. Darker shades of gray indicate higher conservation. **(C)** Tertiary structure of PfEMP1 intracellular domain (ATS) as recently determined using NMR spectroscopy (Mayer *et al*., 2012). The structure reveals a conserved core composed of four alpha helices, which map to the conserved sequence blocks of the WRD-A and WRD-B subdomains as indicated by red bars above alignments. Abbreviations: LCR… low complexity region.

As shown in Table 2, hierarchical clustering supports the previously proposed link between the well-studied *Plasmodium* VSA gene families *var* (encoding PfEMP1 proteins), *SICAvar*, and *surfin/pvstp1*[17]. Unexpectedly, the *surfin/pvstp1* cluster includes two genes from *P. gallinaceum* (which we named *PgSurf1* and *PgSurf2*) that have very similar gene structures to *P. falciparum surfin* genes (Figure 4A), suggesting that SURFINs are conserved outside human malaria parasites. Further up the tree, SURFIN/PvSTP1 proteins first group with *P. knowlesi* SICAvar proteins (28 genes and 214 pseudogenes, MP = 35.7%, Avg.E = 0.013) and then with another large cluster (MP = 1.8%; Avg.E = 1.9) containing the two *P. falciparum* gene families *var* (69 genes, 38 pseudogenes) and *dbl* (4 genes) as well as six other DBL domain-containing proteins, including two MSP3 proteins (PF10_0348 and PF10_0355), MAEBL (PF11_0486), AMA1 (PF11_0344), giant protein Pf332 (PF11_0506), and one protein of unknown function (PFA0665w). Notably, still further up the tree the *surfin*/*pvstp1*/*SICAvar*/*var* gene cluster is joined by the large gene family *pir* (1,814 genes), uniting all major *Plasmodium* VSA gene families except *rifin*/*stevor* in a single gene cluster. However, with only 772 BLAST high-scoring segment pairs (HSPs) in total and an average E-value of 14 the connectivity between *surfin*/*pvstp1*/*SICAvar*/*var* and *pir* was found to be extraordinarily low (Table 2). Because *rifin*/*stevor* genes are not part of this unified VSA gene cluster we conclude that there is no sequence-based evidence supporting a link of *rifin*/*stevor* and *pir*. However, as did others before us [20], we noticed suspicious similarities in some protein features (protein size, positioning of TM segments, secondary structural elements) that make such a link plausible (Additional file 4).

To better understand the nature of the relationship among SURFIN/PvSTP1, SICAvar, PfEMP1, and the DBL-containing proteins, we investigated the sequence similarity matrix of this cluster as well as pairwise BLAST sequence alignments. As revealed by the sequence similarity heat map (Additional file 5), SICAvar and PfEMP1 proteins share no direct pairwise sequence similarity with each other but exhibit weak similarity with SURFIN/PvSTP1 proteins (SICAvar: MP = 36%/E = 0.013; PfEMP1: MP = 17%/E = 0.7). Notably, one *P. knowlesi* SICAvar protein (PKH_081360) and two of the DBL-containing proteins (Pf332 and PFA0665w) stand out by having high sequence similarity not only with paralogs of their respective gene families but also with SURFIN/PvSTP1 proteins (PKH_081360: MP = 94%/E = 4e-13; Pf332: MP = 89%/E = 7e-6; PFA0665w: MP = 89%/E = 0.2). By examining the underlying BLAST alignments we found that BLAST consistently identified HSPs within the C-terminal intracellular regions of proteins, where, in SURFIN/PVSTP1 proteins, the HSPs overlapped with the previously described tryptophan-rich domain (WRD) [17]. In PfEMP1 proteins, the HSPs overlapped with the PfEMP1 intracellular region also known as the acidic terminal sequence (ATS) or VARC [15,39].

Subsequent multiple sequence alignments of SURFIN, PfEMP1 and SICAvar intracellular regions revealed a more complete picture of the relationship of these proteins than described previously [17]. We find that SURFIN WRD has a modular architecture consisting of two distinct and structurally conserved subdomains, which we named WRD-A and WRD-B (Figure 4). WRD-A and WRD-B occur in variable numbers and configurations in different gene families (Figure 4A). The typical WRD consist of one WRD-A (shown in green) and two WRD-B subdomains (shown in blue), which is found in *P. falciparum* (6–7 copies) and *P. gallinaceum* (4–5 copies) SURFIN proteins, the two full-length *P. vivax* PvSTP1 proteins (single copy), and the two *P. falciparum* DBL-containing proteins, including Pf332 (single WRD copy, reported previously) and PFA0665w (1–2 WRD copies, new finding). Where WRDs occur in tandem, the first of the two WRDs is typically better conserved, as illustrated by the partially empty dashed rectangles representing cases where complete WRDs but not individual subdomains reached statistical significance in a HMMER3 search (E ≤ 0.01). In other gene families, WRD is only partially conserved. The typical SICAvar protein contains a single WRD-A but no WRD-B subdomain, while PfEMP1 proteins carry one copy of both WRD-A and WRD-B separated by a ~130 aa long variable region. During our studies an experimentally determined three-dimensional structure of the PfEMP1 intracellular ATS domain became available, which shows that ATS consists of a conserved core composed of four alpha helices [40] (Figure 4C). These four helices map nicely to the two conserved homology blocks corresponding to WRD-A and WRD-B (Figure 4B, red bars above alignments), supporting the biological significance of these alignments.

Notably, we identified at least one *P. knowlesi* SICAvar protein (SICAvar-like gene PKH_081360) with a completely conserved WRD (one WRD-A and two WRD-B), which explains the high BLAST sequence similarity of this protein with *P. falciparum* SURFINs and provides, for the first time, compelling alignment-based evidence that the two antigenically variant gene families *var* and *SICAvar* are evolutionarily linked via shared intracellular WRD domains. A TBLASTN search against the *P. knowlesi* genome using WRD-B of PKH_081360 as query identified one additional SICAvar antigen (PKH_102071, E = 4e-11) carrying a WRD-B subdomain, but this gene appears to be severely truncated (295 aa) and lacks WRD-A, suggesting that PKH_102071 is possibly a pseudogene.

### New members of variant gene families predicted by sequence clustering

Sequence clustering complements consensus-based strategies (using for example Pfam HMMs) for the identification of gene family members. We therefore examined gene clusters of sufficient quality (J ≥ 0.6, Table 1) for the presence of putative new gene family members. Table 3 lists newly predicted members of several *P. falciparum* gene families, including *phist* (4 genes), *hyp4/hyp6* (3 genes), *TSP_1* (2 genes), *msp-7* (2 genes), *msp-3* (1 gene), *etramp* (1 gene), *hyp5* (1 gene), *hyp15* (1 gene), and *hyp10* (1 gene). In none of these cases could we identify obvious cases of misclassifications, suggesting that most of these genes are indeed potential novel members of these gene families.

**Table 3:** **Putative new *P. falciparum *** **gene family members of variant gene families**

**Gene family**	**New member**	**MP#**	**MP %**	**Avg. E-value**	**Avg. PID (Stddev)**	**Current annotation/comment**
*phist*	PF14_0744	21	9.9	0.2	27.6(5.6)	Exported protein, unknown function
	PF14_0745	23	10.9	0.3	24.7(4.7)	Probable protein, unknown function
	MAL8P1.205	40	18.9	0.0002	28.2(4.4)	GEXP13
	PF10_0015^a^	88	41.5	7e-14	35.0(11.9)	Carries acyl-CoA binding domain
*hyp4/hyp6*	PFL2560c	2	12.5	3e-15	46.3(14.4)	Probably truncated pseudogene
	PFI0086w	5	31.3	0.002	29.0(3.7)	Alternative start codon 40 aa downstream
	PF14_0760	4	25.0	0.2	35.7(13.2)	Exported protein, unknown function
TSP_1	MAL8P1.45	48	23.3	7e-17	42.7(14.7)	Conserved protein, unknown function
	PF08_0136b	24	11.7	4e-34	35.3(16.3)	Von Willebrand factor A-domain related
*msp-7*	PF13_0192	14	46.7	0.9	25.7(4.8)	Part of MSP7 gene cluster on chr13
	PF13_0194	3	10	3	28.8(3.3)	Part of MSP7 gene cluster on chr13
*msp-3*	PF10_0351	4	14.3	4	30.4(3.8)	Part of MSP gene cluster on chr10
*etramp*	PFL0065w	2	2.8	9e-15	43.7(6.5)	Conserved protein, unknown function
*hyp5*	PF14_0743	10	100.0	3e-5	31.4(2.5)	Annotated hyp15 protein
*hyp15*	PFB0950w	3	100.0	3e-18	44.5(13.0)	Exported protein, unknown function
*hyp10*	PF08_0001	1	50.0	3	34.6(0.0)	Exported protein, unknown function
*hyp12*	MAL8P1.206	3	100.0	2e-17	47.1(4.9)	Exported protein, unknown function
*hyp17*	PF14_0741	1	50.0	1e-198	91.2(0.0)	Split gene model; merged in PlasmoDB 9.0

Clusters corresponding to variant gene families were searched for *P. falciparum* proteins that are currently not annotated as members of these gene families. Only clusters with a Jaccard index of at least 0.6 were considered in this search (Table 1). Annotated pseudogenes and gene fragment not shown. *MP #* and *MP%* indicate number and percentage of BLAST match pairs between the putative new member and all other proteins in the cluster representing that gene family. *Avg. E-value* and *Avg. PID* denote the average E-value and the average PID of these BLAST match pairs, respectively. ^a^Unusual *phist* gene family member carrying an N-terminal acyl-CoA-binding protein domain (Pfam PF00887).

**Figure 5:**
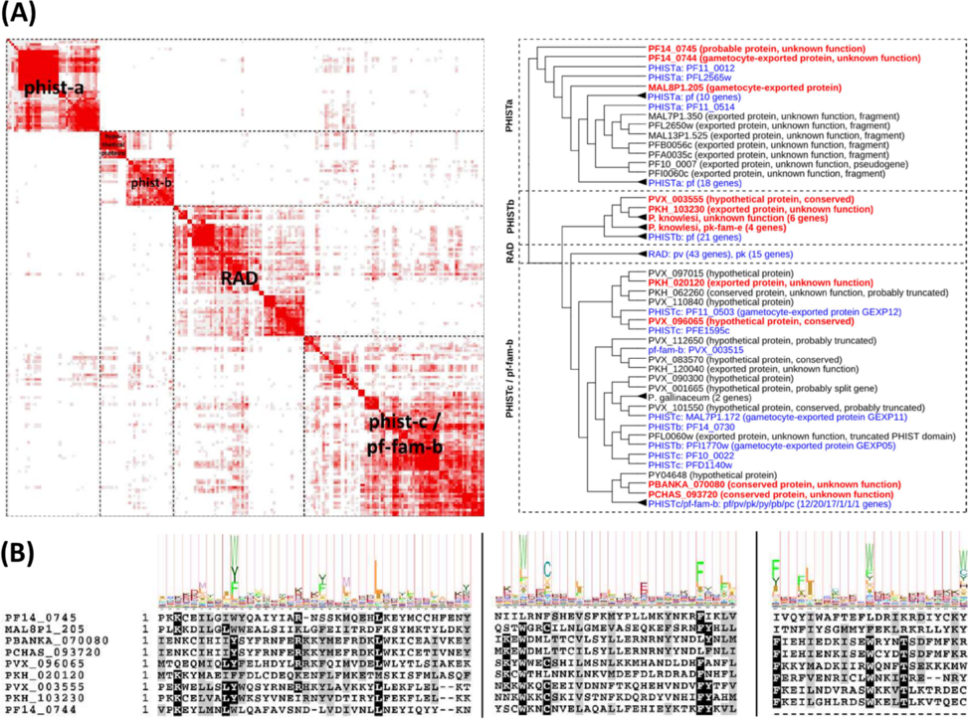
**Predicted new members of the exported gene family *phist *in several *Plasmodium *species, including rodent parasites. (A)** Left: Heat map of pairwise BLAST sequence similarities between all proteins of the PHIST cluster. Shades of red indicate degree of similarity (red = high similarity, white = no similarity). Right: Compressed PHIST dendrogram extracted from the hierarchical tree. Annotated *phist* gene family members indicated in blue. Putative new gene family members shown in red (full-length proteins) or black (annotated pseudogenes or gene fragments). Note the putative new *phist* gene family members in rodent parasites at the bottom. Black triangles indicate collapsed subtrees with numbers of genes contained in this subtree specified in parentheses. **(B)** Top: Pfam family PF09687 (PRESAN domain) sequence logo highlighting conserved key residues of the *phist* gene family. Bottom: Corresponding conserved blocks extracted from a T-coffee multiple sequence alignment of selected putative new PHIST members. Note that these putative new gene family members have most key residues of the PRESAN domain conserved, suggesting that they are likely true members of the diverse *phist* gene family.

To verify the credibility of these predictions, we examined the clustering result of the large and highly divergent *phist* gene family in more detail (Figure 5). *Phist* (Plasmodium helical interspersed subtelomeric) is a large subtelomeric gene family of exported proteins of unknown function with ~70 members in *P. falciparum*, ~40 members in *P. vivax*, and ~27 members in *P. knowlesi*. So far only one gene family member has been identified in each of the three rodent parasite genomes, suggesting extensive radiation of the *phist* gene family in primate parasites [23]. In *P. falciparum*, *phist* has been divided into three subfamilies (*phist-a*, *phist-b*, and *phist-c*). Another chromosome-internally expanded *P. vivax* gene family of 44 genes named *rad* (aka *pv-fam-e*) has later been shown to be closely related to *phist*[10].

In our results, the *phist-a*, *phist-b*, *phist-c*, and *rad* gene families are resolved with high accuracy (J ≥ 0.9) and as expected cluster together into the larger *phist* gene family (Sn = 0.97; Sp = 0.94; J = 0.92) (Table 1 and Figure 5A). *Phist-c* first clusters with *rad* followed by *phist-b* and *phist-a*, which differs from an earlier report suggesting that *phist-a* and *phist-b* form a clade with *phist-c* being the out-group [23]. In total, the *phist* cluster contains 22 full-length proteins that are currently not annotated as *phist* gene family members, including the four genes from *P. falciparum* mentioned earlier (PF14_0744, PF14_0745, MAL8P1.205 (GEXP13), and PF10_0015), two from *P. vivax* (PVX_003555 and PVX_096065), 14 from *P. knowlesi* (including four exported proteins from the *pk-fam-e* gene family that clusters with *phist-b*), and one from each *P. berghei* and *P. chabaudi* (PBANKA_070080 and PCHAS_093720). The two novel rodent parasite *phist* family members cluster with the *phist-c* subfamily and could be interesting targets for experimental studies of PHIST function in the rodent model system. Multiple protein sequence alignment of these putative new *phist* gene family members (Figure 5B) shows that key residues of the PHIST PRESAN domain (PF09687) are well conserved, suggesting that these 10 genes are likely true members of the *phist* gene family. Notably, the PHIST cluster also contains two proteins from *P. gallinaceum*, supporting previous findings that PHIST is conserved in the bird parasite [23]. We conclude that our clustering procedure resolves even challenging gene families correctly and is a successful strategy to identify putative new gene family members in *P. falciparum* and other *Plasmodium* species.

## Discussion

In this study, we classified and investigated VSA and exported gene families across seven *Plasmodium* genomes. This led to several interesting findings, some of which may have application value.

### Conserved PIR proteins are targets for functional studies and vaccine development

The identification of a single ultra-conserved PIR ortholog in each of the PIR-containing *Plasmodium* species (Figure 1) was highly unexpected given that *pir* is the largest and one of the most diverse gene families in *Plasmodium* parasites. Pir gene family sizes range from 68 in *P. knowlesi* to 838 in *P. yoelii*, with amino acid similarities ranging from 30-50% between *P. chabaudi*, *P. yoelii* and *P. berghei* and from 20-30% between *P. chabaudi* and *P. vivax*[41]. The same conserved PIR orthologs were found recently in an independent work studying genetic diversity of the global *P. vivax* population [42]. It was shown that *P. vivax* gene PVX_113230 (one of the five conserved PIR orthologs) is completely invariant across four strains sampled from different sites around the world and also exhibits conserved synteny in rodent malaria parasites. The fact that these highly conserved PIR orthologs could be identified in our study clearly highlights the value of comparative genomics in general and of comparative gene family classification in particular.

The discovery of ultra-conserved PIR orthologs has two important implications. Firstly, these genes can now serve as starting points to elucidate PIR function, which is still unknown. For example, gene knockouts of the conserved PIR orthologs in the more accessible rodent model system may result in important and measurable phenotypes. Alternatively, tagging the conserved PIR orthologs with different fluorescence markers coupled with the manipulation of their expression levels should also help clarifying PIR localization and function. PVX_113230 has a distinct expression profile in erythrocytes relative to most other *vir* genes [43] and therefore may serve a different, ancestral function. If true, insights from the proposed experiments may not be directly applicable to other members of the gene family, but should nevertheless be informative.

Secondly, the finding of a conserved PIR ortholog in *P. vivax* has potential ramifications for *P. vivax* vaccine development. If, like other VIR proteins [19], PVX_113230 is confirmed as surface-exposed antigen, antibodies raised either against PVX_113230 or its hypothetical host receptor(s) could elicit exquisite cross-strain immunity. That parasite genes with limited genetic diversity can be effective vaccine targets was recently demonstrated in *P. falciparum*, where antibodies against an essential and highly conserved ligand for erythrocyte invasion (PfRh5) elicited exquisite neutralizing cross-strain immunity [44,45]. This seemingly paradoxical situation, that a highly conserved parasite protein can be successfully targeted by the host immune system, makes sense if one imagines PfRh5 as functionally important and therefore conserved component of a host-parasite interaction complex. Within such a complex, PfRh5 would not be under direct immune attack because it is efficiently shielded by immunodominant paralogs that act as red herrings or “smoke-screen” [46]. A similar model has been proposed for *rif/stevor* and *surfin* genes in the context of the merozoite invasion process [47]. If this model applies to PIR proteins, then PVX_113230 could be a promising target for the development of a *P. vivax* vaccine.

### Lipid-binding PYST-A proteins potentially involved in cholesterol salvage

Another family of surface antigens that is currently without known function is the large rodent parasite gene family *pyst-a* (named *pc-fam-1* and *pb-fam-1* in *P. chabaudi* and *P. berghei*, respectively). Studies of *pyst-a* expression have shown that the *pyst-a* family member and glutamate-rich protein Pc90 (also known as Pc(em)93, Pc(em)96, and Pch105/RESA [48]) is the immunodominant protein within red blood cell (RBC) membranes and localizes to the cytoplasmic face of the membrane [49,50]. More recently, RBC membrane localization of a *pyst-a* homolog (PBANKA_083680) has also been demonstrated in *P. berghei*[51]. *Pyst-a* genes are concurrently expressed in large numbers without evidence of differential expression in response to different host environments [52], suggesting that altering expression of *pyst-a* gene family members is not an immune evasion strategy of the parasite. Using bioinformatics sequence and structural analysis we predict the existence of a *StAR-related lipid-transfer* (START) domain in *pyst-a* proteins (Figure 2). START is the archetypical domain found in the *steroidogenic acute regulatory* (StAR) protein, which is part of a multi-protein complex termed the “transduceosome” whose function in mammals is to transfer cholesterol across the two mitochondrial membranes and to initiate the first enzymatic step in steroid biosynthesis [53,54]. Cholesterol is also a major lipid fraction in the membrane bilipid layer and is required for membrane genesis. *Plasmodium* parasites cannot synthesize cholesterol *de novo*[55]. It has been shown that host cell membranes of *P. falciparum*-infected erythrocytes show a 50% decrease in the cholesterol/phospholipid ratio compared to uninfected cells [56]. In addition, malaria infections result in hypocholesterolaemic conditions [57]. Together these observations suggest that the parasite scavenges required cholesterol from both host erythrocyte membranes and plasma. How this is accomplished by the parasite is currently not understood. Based on the presence of a cholesterol-binding START domain in RBC membrane-localized PYST-A proteins we hypothesize that PYST-A is part of a molecular machinery that diverts cholesterol away from erythrocyte membranes to fuel parasite growth within erythrocytes. Notably, *P. chabaudi* PcEMA1 proteins could constitute a second component of this proposed cholesterol import machinery. We found five PC-FAM-1 proteins (PCHAS_060180, PCHAS_140150, PCHAS_137050, PCHAS_110050, and PCHAS_042050) that share sequence similarity with both PYST-A (C-terminal) and PcEMA1 proteins (N-terminal). We could not find obvious problems with the gene models of these five proteins, suggesting that they are genuine PYST-A/PcEMA1 protein hybrids. Co-occurrence of PYST-A and PcEMA1 domains within the same polypeptide suggests physical interaction between PYST-A and PcEMA1 proteins. This possibility is supported by the fact that PcEMA1 proteins also localize to the cytoplasmic face of the RBC membrane [58]. It remains to be shown why rodent malaria parasites maintain so many PYST-A proteins compared to primate malaria parasites. PYST-A proteins are probably immunogenic in rodent but not primate parasites, putting PYST-A proteins under strong selective pressure to diversify. Alternatively, massive *pyst-a* gene amplification could increase the efficiency of cholesterol salvage, probably satisfying an elevated need for cholesterol in rodent parasites or compensating for lower cholesterol levels in rodent blood.

### Pfmc-2TM – conserved beyond *P. falciparum*

Another interesting erythrocyte surface-expressed gene family of currently unknown function is the *P. falciparum* gene family *Pfmc-2TM*. The 12 members of the *Pfmc-2TM* gene family encode putative functional and highly similar (avg. global pairwise PID = 76%, range 61-96%) basic membrane proteins that, based on structural features, are grouped within the larger 2TM superfamily. The 2TM superfamily consists of 200–300 aa long proteins that on their first exon carry a signal peptide followed by a N-terminal PEXEL motif and two C-terminal TM domains on their second exon. Besides *Pfmc-2TM*, the 2TM superfamily includes *rif/stevor* as well as genes in *P. knowlesi*, *P. vivax*, and the rodent parasite genomes that share similar architectural features [59]. It is important to note that, other than *pir*, the large 2TM superfamily is currently united solely based on gene structural features and not sequence similarity; in fact, to date no PfMC-2TM sequence homologs have been identified in *P. falciparum* or any other species. PfMC-2TM proteins have been localized to the Maurer’s clefts [18,36], the parasitophorous vacuole, parasitophorous vacuole membrane [36], and the erythrocyte surface [60]. At this latter location, 2TM proteins including PfMC-2TM are thought to interact with the host immune system through a surface-exposed and hypervariable loop region that is located between the two TM domains [60]. PfMC-2TM proteins are most highly expressed in the mid-trophozoite stage and have been shown to undergo expression switching, suggesting that the *Pfmc-2TM* gene family plays a role in antigenic variation [18,35].

We provide first evidence that the *Pfmc-2TM* gene family has additional members both within and outside the *P. falciparum* species (Figure 3). Experimental clues for the existence of potential *Pfmc-2TM* homologs in the rodent *Plasmodium* genome already existed, but the identity of these genes remained unknown [36]. Furthermore, we showed that PfMC-2TM proteins contain a conserved and putative functional domain located between PEXEL and the two TM domains, which we named MC-TYR. These two findings have important implications for future studies of PfMC-2TM function. Experimental double-knockout studies can now be attempted in rodent parasites on the two *Pfmc-2TM* gene family members to see if they produce an observable phenotype. Given the reduced number of *Pfmc-2TM* gene family members in rodent malaria parasites compared to *P. falciparum* and the more tractable rodent model system, such studies should now be feasible. Two additional observations suggest that *hyp2*, *hyp8*, and the six rodent malaria proteins are genuine members of the extended *Pfmc-2TM* gene family. First, all but three of the putative new *Pfmc-2TM* members (PCHAS_110090, PFB0926c, and PFB0970c) have a similar basic isoelectric point (9.2-10.5 pH) as known PfMC-2TM proteins. Second, all newly identified members that have two TM domains carry a single proline residue within the second TM domain, another known characteristic of PfMC-2TM proteins [18]. Proline residues internal to helices are often found in transporters, channels, and receptors, and tend to be conserved between homologous proteins. In contrast, we did not observe conservation of the two cysteine residues preceding the TM domains [18]. Also the fact that no TM domains are present in three of the proteins carrying MC-TYR is intriguing and suggests that the presence of two TM domains is not essential for a possible MC-TYR function. It is also noteworthy that most of the *hyp2* and *hyp8* gene family members are more broadly expressed throughout the intra-erythrocytic life cycle than PfMC-2TM proteins, showing expression not only in the mid-trophozoite stage but also in early ring and schizont stages (PFB0926c, PFB0970c, MAL13P1.61, and PFA0670c) as well as in gametocytes (*hyp8*) (PlasmoDB 9.0). This suggests that the function of MC-TYR containing proteins is not restricted to trophozoites. Our clustering results do not provide evidence for a link between *Pfmc-2TM* and the *pyst-b* gene family as has been proposed previously based on gene structural features [18].

### WRD as missing link between major *Plasmodium* VSA gene families

Besides the large 2TM superfamily, malaria parasites express additional VSA gene families on cell surfaces. Among them are the *var* gene family in *P. falciparum* and *SICAvar* in *P. knowlesi*, both of which encode proteins expressed at the erythrocyte surface and are known to undergo antigenic variation. SURFIN, a family of 10 *P. falciparum* proteins, has been shown to be expressed on the surfaces of both infected erythrocytes and merozoites [17]. By examining intracellular regions of co-clustered SURFINs, PfEMP1 and SICAvar proteins, we now better understand the sequence conservation pattern present among these major *Plasmodium* VSAs (Figure 4). We found that SURFIN proteins contain twice as many WRDs as reported previously [17] and that each WRD consists of three conserved blocks. The first block, named WRD-A (40–60 aa), is found once in each of the WRDs, whereas block two and three represent two copies of a second subdomain that we named WRD-B (40–50 aa). Winter *et al*. previously described the conservation pattern among WRD-containing proteins in terms of shared conserved elements denoted S1, S2, S2*, S2**, and PEXEL-like motif [17]. Compared to this earlier study, we find that WRD-A corresponds to segment S1 plus five additional C-terminal amino acids in WRD-A, which map to the second alpha helix of the protein structure (Figure 4B). WRD-B corresponds roughly to the C-terminal half of segment S2, and segment S2* (featuring a duplication within antigen Pf332) is in fact the first of the two WRD-B copies found within this protein. The short S2** subsegment, indicating another duplication at the end of each WRD in SURFIN 4.2, is in fact the most conserved part of the second copy of the WRD-B subdomain in this protein. The PEXEL-like motif localized within the S1 segment was also found to be well conserved in our study (Figure 4B). Interestingly, this PEXEL-like motif currently has no correspondence in the reported tertiary structure (Figure 4B and Figure 4C), which might be explained by the fact that the ATS-Core deletion construct that was used to determine the ATS structure lacked this PEXEL-like motif [40]. It thus remains to be shown if the PEXEL-like motif is in fact part of ATS-Core or serves another conserved function in WRDs.

Importantly, our data provides the first conclusive alignment-based evidence that the two major antigenically variant gene families in *P. falciparum* (*var*) and in *P. knowlesi* (*SICAvar*) share common evolutionary origin, substantiating the usefulness of the *P. knowlesi*-rhesus monkey model for the *in vivo* study of *P. falciparum* antigenic variation. In 2005, Winter *et al*. provided the first bioinformatics evidence that PfEMP1 and SICAvar proteins are probably evolutionarily linked through their intracellular domains, which share partial sequence similarity with the intracellular tryptophan-rich domain (WRD) of SURFIN proteins [17]. Short conserved sequence motifs have also been identified in *var* and *SICAvar* introns [11] as well as in extracellular regions of these proteins [61]. However, at present the picture remains highly fragmented and reported sequence similarity of intracellular regions between PfEMP1 and SICAvar is essentially limited to a highly conserved six amino acid long PEXEL-like motif, questioning the robustness of these observations. Our study provides new evidence for common ancestry of PfEMP1 and SICAvar, which rests on three critical observations. Firstly, not only WRD-B (formerly segment S2) but also WRD-A (formerly segment S1) is entirely conserved in PfEMP1 proteins. Secondly, at least one SICAvar protein (PKH_081360) has a completely conserved WRD, consisting of one WRD-A and two WRD-B subdomains. Thirdly, complete WRDs are conserved in at least two other *P. falciparum* proteins (Pf332 and PFA0665w), which are clearly related to PfEMP1 proteins through N-terminal DBL domains. Why were these sequence similarities not detected previously? Unlike SURFINs, PfEMP1 proteins have WRD-A and WRD-B separated by a larger (~130 aa) variable insertion, which in the protein structure does not adopt a stable fold [40]. Similarly, compared to SURFINs, PfEMP1 proteins carry additional insertions both within WRD-A (~20 aa insertion) and WRD-B (~3 aa insertion) (Figure 4B). In WRD-A, this insertion splits the highly conserved PEXEL-like motif from the rest of the WRD-A subdomain (Figure 4B). The presence of these insertions in PfEMP1 proteins combined with the fact that WRD-B is absent in all but one SICAvar protein likely thwarted earlier attempts to find significant sequence conservation between PfEMP1 and SICAvar proteins [17,20]. It is not too surprising that larger blocks of conserved sequence between PfEMP1 and SICAvar are confined to intracellular regions as extracellular regions are likely under strong diversifying selection pressure from the host immune system. Consistent with earlier reports, we did not find evidence that WRD or any of its subdomains is conserved within the large *pir* gene superfamily of *Plasmodium* surface antigens [17].

### What is the function of WRD?

Despite this now much improved picture of the conservation structure of PfEMP1, SICAvar, and SURFIN intracellular domains, many fundamental questions remain. Why do SURFINs have multiple copies of complete WRDs while most other gene families carry only a single WRD? What is the functional significance of having two WRD-B subdomains (SURFINs and Pf332) *vs.* having only one WRD-B subdomain (PfEMP1)? Why is WRD-B found in only a single SICAvar protein (PKH_081360) while all other members of the *SICAvar* gene family carry only the WRD-A subdomain? Unfortunately, the molecular function of WRD is currently not well understood, so these questions are difficult to answer. Studies have shown that ATS of PfEMP1 interacts either directly or indirectly via the knob-associated histidine-rich protein (KAHRP) with erythrocyte membrane skeletal proteins, including actin and spectrin [39,62-65]. ATS is thus believed to anchor PfEMP1 at the membrane of infected RBCs, which strengthens and stabilizes the roots of PfEMP1-endothelial receptor interaction. Similarly, Pf332, which contains a single complete WRD near its C-terminal end, was found to contribute to the overall deformability of *P. falciparum*-infected erythrocytes through anchoring and scaffolding [66,67], probably through WRD-mediated binding of Pf332 to actin [68]. It seems therefore plausible that the main function of WRD is to provide a scaffold for anchoring surface antigens like PfEMP1 to the RBC cytoskeleton. The function of having many WRDs resulting from gene family expansion and/or intra-protein domain amplification is then probably to increase the strength of this scaffold. This would explain why particularly *P. falciparum*, which compared to other malaria parasites has strong cytoadherence properties, expresses so many WRDs in form of the *surfin* gene family. SURFINs have not yet been implicated in anchoring surface antigens to the erythrocyte cytoskeleton, but we think that this is a plausible hypothesis that should be tested experimentally. It is also worth mentioning that the ABBABBABB-like repeat structure of the SURFIN WRDs is reminiscent of similar repeats present in other cytoskeleton-binding proteins, including the vertebrate titin and the invertebrate twitchin muscle proteins [69]. Regardless of the exact function of WRD, its modular nature suggests that different proteins carrying distinct WRD subdomains act in concert to perform this function. *P. knowlesi* protein PKH_081360, which appears to be the only functional protein in the *P. knowlesi* genome carrying both WRD-A and WRD-B, thus probably interacts physically with other, WRD-A-containing SICAvar proteins to anchor SICAvar to the cytoskeleton. With the refined domain structure presented here it should now be possible to test this prediction experimentally. Ultimately, such experiments will hopefully expand our knowledge of how *P. falciparum* accomplishes cytoadherence at the molecular level, which is responsible for much of the virulence of this parasite.

## Conclusions

Sensitive clustering of protein sequences followed by manual examination of clustering results revealed many new insights into variant surface antigens of malaria parasites, including atypical and new gene family members, novel domains, new protein function predictions, and a better understanding of the evolutionary relationship and origin of these important proteins. The findings presented in this work can now jump-start follow-up experimental research around the world, most importantly to elucidate the function of PVX_113230 and to explore its potential value as *P. vivax* vaccine target.

## Methods

### Protein sequence data set

The raw protein sequence data set included 259,778 protein sequences from 18 species, including seven *Plasmodium* species (*P. falciparum*, *P. vivax*, *P. knowlesi*, *P. yoelii*, *P. berghei*, *P. chabaudi*, *P. gallinaceum*), three related Apicomplexan parasites (*Theileria parva*, *Toxoplasma gondii*, *Cryptosporidium parvum*), seven other, well-studied model organisms (*Drosophila melanogaster*, *Caenorhabditis elegans*, *Monosiga brevicollis*, *Saccharomyces cerevisiae*, *Arabidopsis thaliana*, *Chlamydomonas reinhardtii*, *Escherichia coli*), and human. Protein sequences were obtained from various sources as summarized in Additional file 6. The rationale for including additional species besides *Plasmodium* was that on the one hand we hoped to improve the sensitivity of the clustering algorithm through the transitivity principle and that on the other hand we expected that functional insights could be gleaned from functionally characterized non-*Plasmodium* proteins that cluster with uncharacterized *Plasmodium* proteins.

### *P. gallinaceum gene prediction*

Because gene annotations for *P. gallinaceum* were not available when this study was initiated, *P. gallinaceum* protein-coding genes were predicted using our own homology-based gene predictor genBlastG (version 1.28) [70]. *P. gallinaceum* supercontigs (4,996) were obtained from the Sanger FTP site (http://sanger.ac.uk/pub/pathogens/Plasmodium/gallinaceum/) and annotated with genBlastG using protein sequences from the well-curated *P. falciparum* gene set (PlasmoDB 6.0) as query (parameters: -c 0.5 -s 0 -r 1 -gff -pro -b). This resulted in 3,141 predicted *P. gallinaceum* protein-coding gene models. It is important to note that this *P. gallinaceum* gene set was created solely for the purpose of comparative gene family classification and should be considered highly preliminary. While *P. gallinaceum* protein-coding genes with sufficient sequence similarity to *P. falciparum* proteins are expected to be well represented, genes or exons with low similarity might be absent or mispredicted. Also, the currently low sequencing coverage of the *P. gallinaceum* genome (three-fold) means that many gene models will be missing.

### Gene model improvement

Besides predicting gene models for *P. gallinaceum*, we also improved existing gene model annotations for the genomes of *P. vivax*, *P. knowlesi*, *P. chabaudi*, *P. berghei*, and *P. yoelii* using a previously developed homology-based gene model improvement pipeline [12,71]. Briefly, protein sequences of all protein-coding *P. falciparum* genes were used as query to run both genBlastG [70] and GeneWise [72] against the other *Plasmodium* genomes. To ensure the quality of predicted gene models, only predictions that encoded for protein sequences with high global sequence identity (PID > = 60) with the query gene were kept. If multiple predictions overlapped by more than 5% of their coding exons, only the prediction with the highest PID to the query was kept (filtration step). In a subsequent merging step, predicted and existing gene models were merged into a hybrid gene set, retaining predictions that (a) did not overlap with existing gene models or (b) showed a PID improvement of at least 5% over overlapping existing gene models. As in the filtration step, existing and predicted gene modes were considered as overlapping if more than 5% of their coding exons overlapped. This hybrid gene set then served as the basis for the subsequent protein sequence clustering step.

### Hierarchical clustering

The raw set of 259,778 protein sequences was filtered to retain only longest isoforms, which reduced the number of protein sequences to 171,476. Low-complexity regions were masked with TANTAN [73] (parameters: -p –s 0.99 –r 0.005) before BLAST analysis because low-complexity filtering as performed by BLAST was found to be insufficient to mask more complex repeats, causing clustering of non-homologous proteins. NCBI BLASTP version 2.2.25+ (http://blast.ncbi.nlm.nih.gov) (parameters: -evalue 100 -num_descriptions 2000 -outfmt 6 -word_size 2 -lcase_masking) was then used to compare protein sequences in an all-*vs.*-all pairwise manner, resulting in a total of 24,206,683 HSPs. HSPs were then filtered to retain only the best HSP (= lowest E-value) per protein sequence pair, resulting in 12,224,106 symmetrified best HSPs. E-values were transformed into positive dissimilarity values for hierarchical clustering using the formula 200-min(200, -log_10_(E-value/100)), whereas E-values of 0 were assigned the minimum dissimilarity value of 0. Hierarchical clustering was performed using MC-UPGMA (version 1.0.0) [28] obtained from http://www.protonet.cs.huji.ac.il/mcupgma/ (parameters: -max_distance = 200 -iterations = 100).

### Cluster extraction

**Figure 6:**
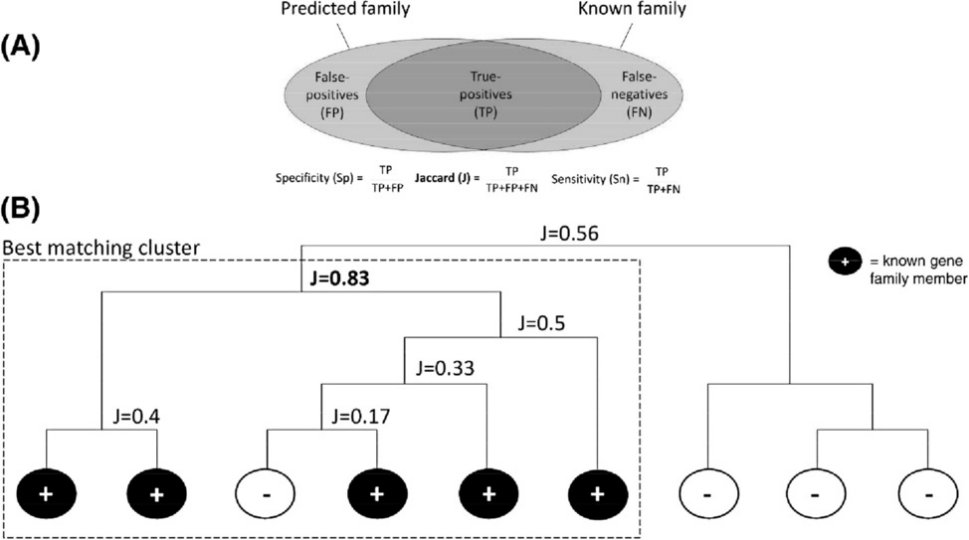
**Gene family classification strategy. (A)** Jaccard index (J) as measure of overlap between a trusted reference gene family (right ellipse) and a predicted gene family (left ellipse). **(B)** Proteins of a known reference gene family (black) are hierarchically clustered with all other proteins of one ore more species (white). From the resulting hierarchical tree the best matching cluster (= sub-tree with the highest Jaccard index, dashed rectangle, here J = 0.83) is extracted to represent the gene family. All proteins in this cluster will be predicted as members of this gene family.

Cluster extraction from the hierarchical tree was performed using custom Perl scripts (Figure 6). Starting at a leaf node corresponding to a known gene family member, we moved up the hierarchical tree until specificity (= TP/(TP + FP)) dropped below 0.1 or sensitivity (= TP/(TP + FN)) reached the maximum value of 1.0 (*i.e.* all known family members are contained within this subtree). For each internal node visited during this bottom-up tree traversal we computed and noted its Jaccard index (= TP/(TP + FP + FN)). The above procedure was repeated for each known member of a gene family. Finally, the internal node with the highest Jaccard index was returned as representative gene cluster for this gene family. Annotated pseudogenes or proteins of non-reference species were not considered when computing specificity, sensitivity, and Jaccard indices. Defined reference species for each gene family are shown in Table 1 and correspond to species that have gene numbers shown in parentheses.

### PIR conservation analysis

For each of the five *pir* subfamilies (*bir*, *cir*, *yir*, *kir*, *vir*), all members of a subfamily were globally aligned to all members of the other four subfamilies. Global protein sequence alignment was performed with ClustalW (version 1.83) [74] using the default BLOSUM matrix and default parameters. The distribution of global percent identity (PID) values shown in Figure 1B was generated using the ‘boxplot’ function of R with the range parameter set to 2.5. The multiple sequence alignment shown in Figure 1A was computed online at http://tcoffee.crg.cat using T-Coffee with default parameters [75]. The alignment was then pruned and formatted with GeneDoc 2.7 [76].

### Pyst-a function and structure prediction

Domain annotations shown in Figure 2A were generated with HMMER3 [77] (http://hmmer.janelia.org/) using HMMs downloaded from SUPERFAMILY version 1.75 [78] (http://supfam.cs.bris.ac.uk/SUPERFAMILY/downloads.html#Models) and visualized with FeatureStack [79]. Downloaded HMMs were first converted into a HMM database using *hmmpress* (default parameters) and then searched against *Plasmodium* protein sequences using *hmmscan* (default parameters). The protein structure prediction of PF14_0604 was generated using I-TASSER server [80,81] with default parameters. TM-score, RMSD, and PID correspond to values outputted by I-TASSER, which used TM-align to compute these values [82]. Protein sequence alignment and secondary structure prediction were also obtained from the I-TASSER output. Both predicted and template protein structures were rendered with PyMOL (The PyMOL Molecular Graphics System, Version 1.3, Schrödinger, LLC).

### Identification and phylogenetic analysis of MC-TYR

PfMC-2TM, HYP2 and HYP8 protein sequences clustering together in the hierarchical tree (Figure 4) were aligned using the T-Coffee web service [75]. The MC-TYR domain was identified by manual inspection of the resulting multiple sequence alignment. Transmembrane domains were predicted using the TMHMM standalone version (v2.0c) [83] and the EBI Phobius Web server [37] (http://www.ebi.ac.uk/Tools/pfa/phobius). Coloring of the MC-TYR multiple sequence alignment was performed with GeneDoc [76] using the ‘Shade 4 Levels’ option. The secondary structure of MC-TYR was predicted with Jpred 3 [84] using the multiple sequence alignment of Figure 4B as input. The phylogenetic tree shown in Figure 3 was produced with MEGA5 [85] using maximum likelihood and 100 bootstrap iterations.

### Identification of WRD-A and WRD-B subdomains

Guided by local BLAST sequence similarities we compiled a hand-curated set of partial SURFIN, PvSTP1, SICAvar, PfEMP1, and DBL-containing protein sequences. These sequences were aligned using PSI-Coffee from the T-Coffee web site [75] with default parameters. Resulting multiple sequence alignments were manually curated with GeneDoc 2.7 [76] and poorly aligned sequences were removed. Curated multiple sequence alignments representing WRD-A (Additional file 7, 19 sequences) and WRD-B (Additional file 8, 32 sequences) subdomains were then converted into Stockholm format at http://myhits.isb-sib.ch/cgi-bin/reformat and used as input for HMMER3 searches (default parameters). We also hand-curated a multiple sequence alignment representing the complete WRD consisting of one WRD-A and two WRD-B subdomains (Additional file 9). HMMER3 predictions were then visualized on top of gene models (Figure 4A) using FeatureStack [79]. Only matches with an E-value of 0.01 or lower are shown. TM domains were identified with TMHMM (v2.0c) [83]. Domain matches in intracellular regions of proteins shown in Figure 4A were also identified with HMMER3 using Pfam v26.0 domains as input (E-Value ≤ 1e-10) [86]. Annotated domains correspond to Pfam entries PF12887 (SICA_alpha), PF12878 (SICA_beta), and PF05424 (Duffy_binding). Gene structures of *PgSurf1* and *PgSurf2* were manually curated based on the presence of two large open reading frames in both genes, representing exon 1 and exon 2. Predicted protein sequences of PgSURF1 and PgSURF2 are provided in Additional file 10.

### Data access

We set up a project Web page (http://genome.sfu.ca/projects/gfc-plasmodium) providing clustering results for all *Plasmodium* gene families examined in this study. For each gene family, this data includes: cluster dendrogram (Newick format); sequence similarity matrix (tab-delimited format); list of protein accessions and descriptions (tab-delimited format); protein sequences (multi-fasta format); and images of annotated gene models in form of both a static and an interactive HTML page. These images also contain cluster dendrograms and intraerythrocytic expression profiles for *P. falciparum* and *P. vivax* proteins.

## Competing interests

The authors declare that they have no competing interest.

## Authors’ contributions

NC and CF conceived the study. CF carried out data analysis and interpretation. CF and NC wrote the manuscript. All authors read and approved the final manuscript.

## Supplementary Material

Additional file 1 **Phylogenetic tree of selected malaria parasite species.** Symbols next to species names indicate infected host species, including human, monkeys, rodents, and birds. The phylogenetic tree is reproduced from [87] and inferred from partial mitochondrial genomes (5,580 bp). Numbers above branch points represent posterior probabilities in percent, and the scale bar represents the number of nucleotide substitutions per site. The avian Haemosporida *Leucocytozoon sabrazesi* was used as out-group. Click here for file

Additional file 2 **Extended version of Table** 1** with gene numbers from additional species.** Click here for file

Additional file 3 **Subtree of the *vir *** **gene family classification result, highlighting gene models and placement of the conserved *pir *** **orthologs.** Click here for file

Additional file 4 Similarities of protein secondary structural features between two PIR and RIFIN proteins. Click here for file

Additional file 5 **Sequence similarity heat-map of all SURFIN, PfEMP1, and SICAvar proteins, pointing out atypical gene family members with high cross-family similarity.** Additional file 6 lists all species and data sources of the proteins classified in this study. Click here for file

Additional file 6 Species and data sources of the proteins classified in this study. Click here for file

Additional file 7 Multiple sequence alignment underlying the WRD-A domain. Click here for file

Additional file 8 Multiple sequence alignment underlying the WRD-B domain. Click here for file

Additional file 9 Multiple sequence alignment underlying the complete WRD domain. Click here for file

Additional file 10 **Protein sequences of the two newly discovered SURFIN proteins in *P. gallinaceum *** **(PgSURF1 and PgSURF2).** Click here for file
