# Supplementary figures and images for: Variant surface antigens of malaria parasites: functional and evolutionary insights from comparative gene family classification and analysis
Source: BMC Genomics. 2013 Jun 27;14:427. doi: 10.1186/1471-2164-14-427 (PMC3747859; doi:10.1186/1471-2164-14-427)

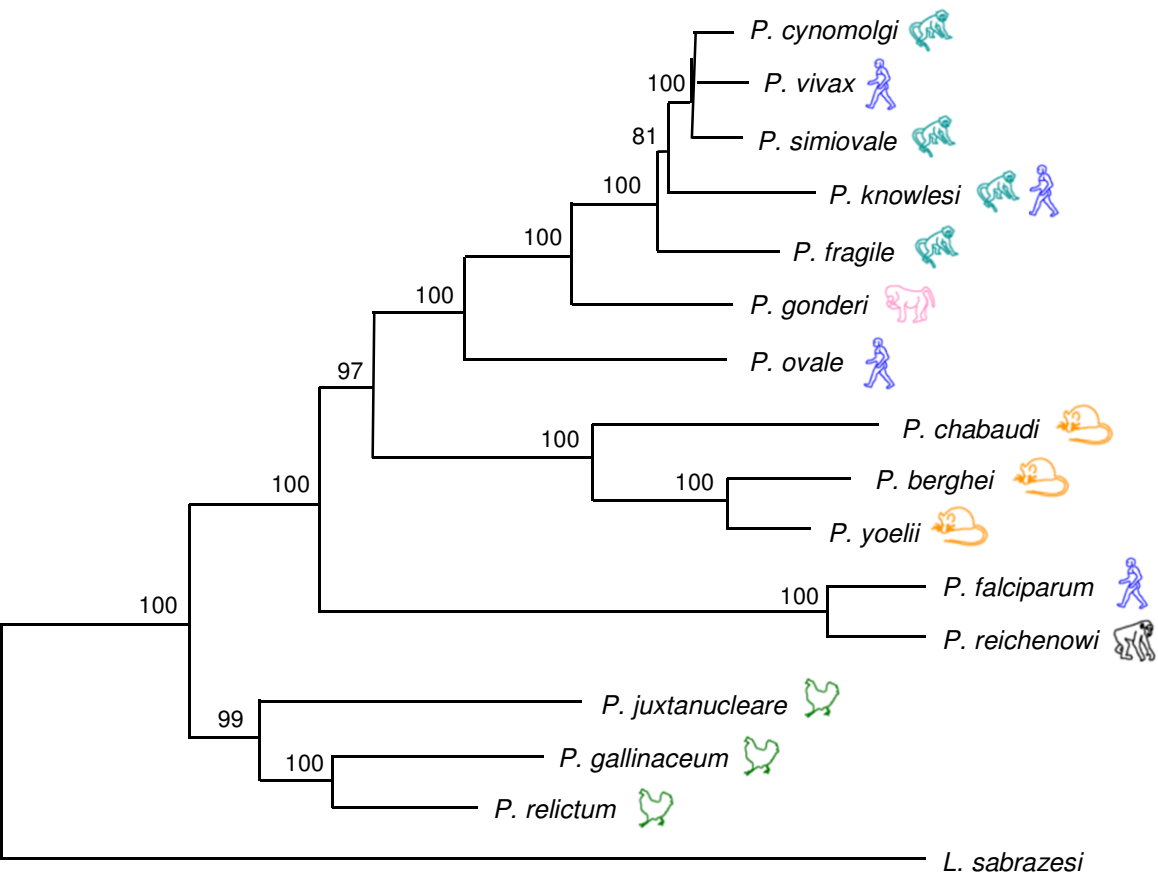

0.02

Supplement: Additional file 1 — Phylogenetic tree of selected malaria parasite species. Symbols next to species names indicate infected host species, including human, monkeys, rodents, and birds. The phylogenetic tree is reproduced from [87] and inferred from partial mitochondrial genomes (5,580 bp). Numbers above branch points represent posterior probabilities in percent, and the scale bar represents the number of nucleotide substitutions per site. The avian Haemosporida Leucocytozoon sabrazesi was used as out-group. [file 1471-2164-14-427-S1.pdf]

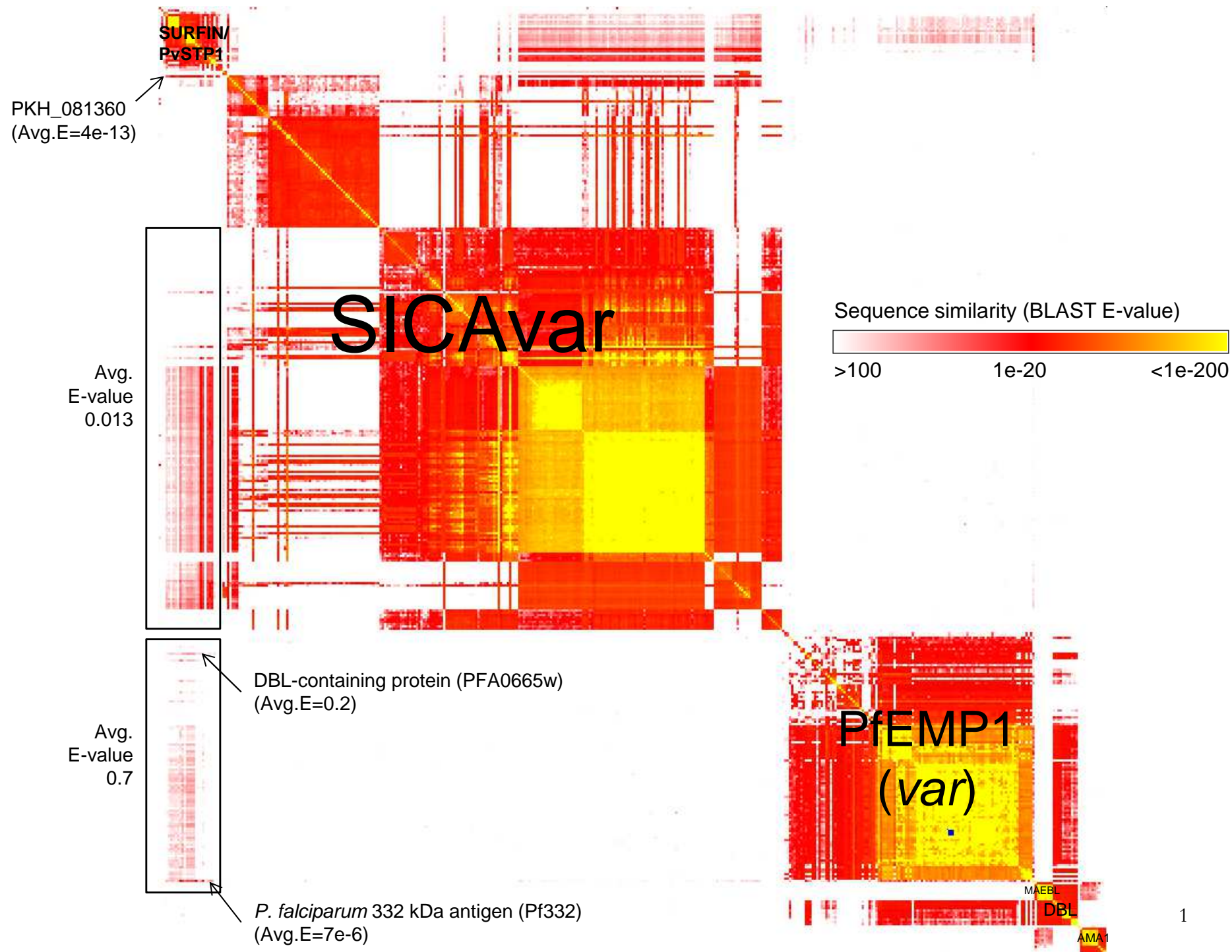

Supplement: Additional file 5 — Sequence similarity heat-map of all SURFIN, PfEMP1, and SICAvar proteins, pointing out atypical gene family members with high cross-family similarity. Additional file 6 lists all species and data sources of the proteins classified in this study. [file 1471-2164-14-427-S5.pdf]
