# Supplementary material for: Variant surface antigens of malaria parasites: functional and evolutionary insights from comparative gene family classification and analysis
Source: BMC Genomics. 2013 Jun 27;14:427. doi: 10.1186/1471-2164-14-427 (PMC3747859; doi:10.1186/1471-2164-14-427)
Supplement: Additional file 3 — Subtree of the vir gene family classification result, highlighting gene models and placement of the conserved pir orthologs. [file 1471-2164-14-427-S3.pdf]

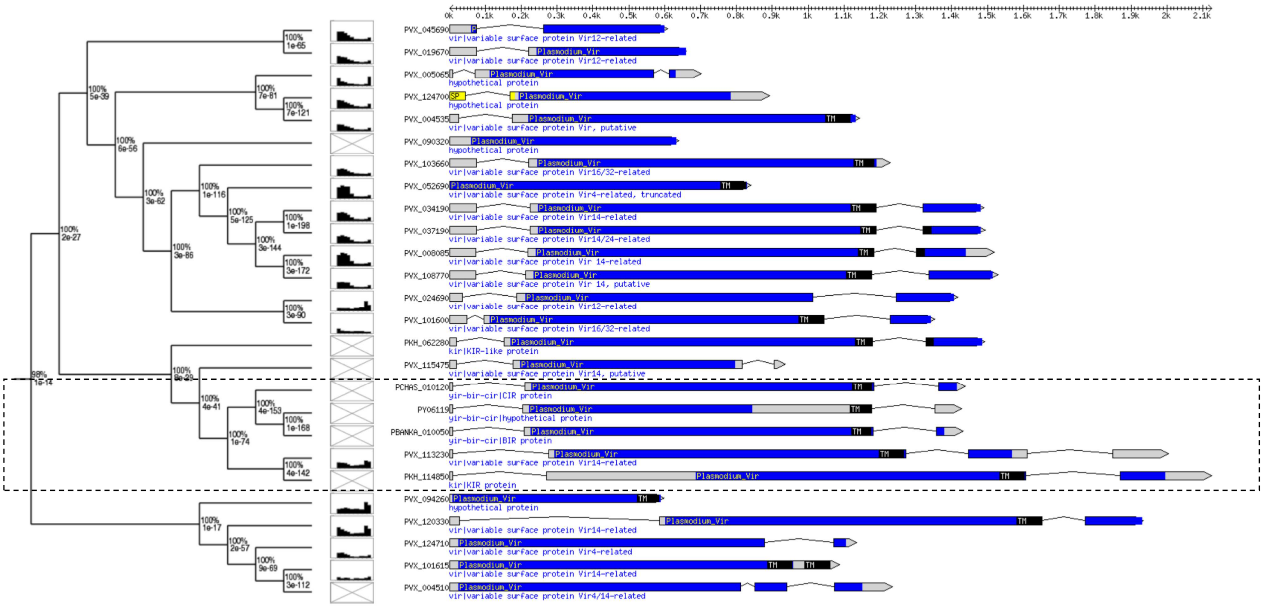

Additional File 3: Sub-tree of the larger *vir/kir* gene cluster showing the placement of the five conserved PIR orthologs. The five conserved *pir* genes (PCHAS_010120, PBANKA_010050, PY06119, PVX_113230 and PKH_114850) are marked with a dashed rectangle. The closest *vir* genes clustering with these five genes are PVX_115475 and PVX_113230, two annotated members of the *vir14* gene family. PVX_115475 has a suspiciously truncated gene structure compared to its closest homologs and other *vir14* genes, suggesting that this gene model is currently mispredicted. The complete *vir/kir* gene cluster can be found at http://genome.sfu.ca/projects/gfc-plasmodium/clusters/vir-kir/static.html.
