# Supplementary material for: Variant surface antigens of malaria parasites: functional and evolutionary insights from comparative gene family classification and analysis
Source: BMC Genomics. 2013 Jun 27;14:427. doi: 10.1186/1471-2164-14-427 (PMC3747859; doi:10.1186/1471-2164-14-427)
Supplement: Additional file 4 — Similarities of protein secondary structural features between two PIR and RIFIN proteins. [file 1471-2164-14-427-S4.pdf]

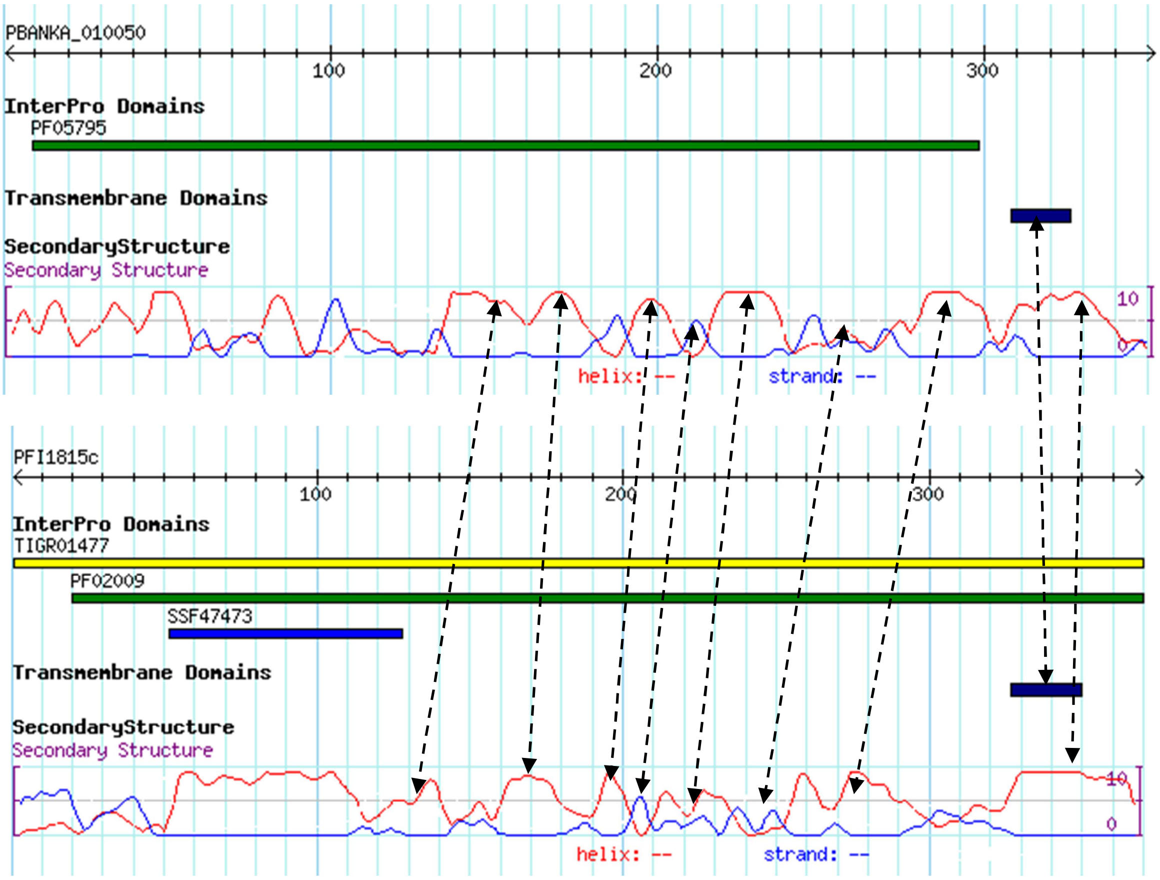

Additional File 4: PIR and RIFIN/STEVOR proteins share similar protein features, suggesting an evolutionary link between these two VSA gene families. The upper half shows the annotated protein sequence of the ultra-conserved *P. berghei* BIR protein PBANKA_010050. The lower half shows *P. falciparum* RIFIN protein PFI1815c. PFI1815c is the best hit in the *P. falciparum* proteome (E=0.00016) when searched with Pfam HMM model *Plasmodium_Vir* (PF05795) using Hmmer3. The two proteins have similar length (~350 aa), have both a C-terminal transmembrane domain, and share similarity in their predicted secondary structural elements, suggesting similar 3D structures, probably of an "Armadillo-like" fold (Bultrini *et al*., 2009). Similarity of secondary structural elements breaks down at the N-terminus, but this could be due in part to the presence of an additional signal peptide and export motif in RIFIN proteins. The figure is based on screenshots taken from PlasmoDB (http://plasmodb.org/).
