# Supplementary material for: Variant surface antigens of malaria parasites: functional and evolutionary insights from comparative gene family classification and analysis
Source: BMC Genomics. 2013 Jun 27;14:427. doi: 10.1186/1471-2164-14-427 (PMC3747859; doi:10.1186/1471-2164-14-427)
Supplement: Additional file 6 — Species and data sources of the proteins classified in this study. [file 1471-2164-14-427-S6.pdf]

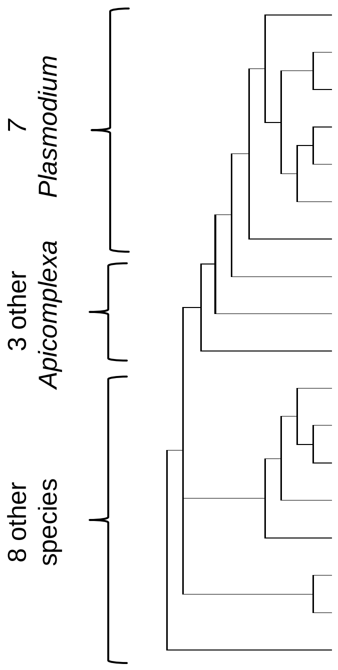

Species	Source	No. proteins	No. longest isoforms
Plasmodium falciparum	PlasmoDB 7.0	5,491	5,458
Plasmodium vivax	PlasmoDB 7.0, genBlastG-improved	5,493	5,493
Plasmodium knowlesi	PlasmoDB 7.0, genBlastG-improved	5,217	5,217
Plasmodium yoelii	PlasmoDB 7.0, genBlastG-improved	7,777	7,777
Plasmodium berghei	PlasmoDB 7.0, genBlastG-improved	4,906	4,906
Plasmodium chabaudi	PlasmoDB 7.0, genBlastG-improved	5,134	5,134
Plasmodium gallinaceum	WTSI, genBlastG-annotated	3,141	3,141
Theileria parva	PiroplasmaDB v1.0	4,082	4,082
Toxoplasma gondii	ToxoDB v6.4	7,940	7,940
Cryptosporidium parvum	CryptoDB 4.4	3,805	3,805
Homo sapiens	Ensembl release 62	88,237	22,111
Drosophila melanogaster	Flybase (FB2011_05)	23,605	14,013
Caenorhabditis elegans	Wormbase WS224	25,010	20,429
Monosiga brevicollis	NCBI genomes	9,203	9,203
Saccharomyces cerevisiae	SGD (June 6, 2011)	6,717	6,717
Arabidopsis thaliana	PlantGDB release 183	35,386	27,416
Chlamydomonas reinhardtii	NCBI genomes	14,489	14,489
Escherichia coli	NCBI Genomes	4,145	4,145
**Total**		**259,778**	**171,476**

Additional File 6: List of the 18 species and number of proteins per species included in the protein sequence data set. The dendrogram to the left shows the phylogenetic relationship of the species and was compiled from the tree of life Web site (http://tolweb.org). WTSI… Wellcome Trust Sanger Institute (http://www.sanger.ac.uk/resources/downloads/protozoa/plasmodium-gallinaceum.html).
